# Coronal and Root Caries in Older Adults: Associated Factors, Quality of Life Impact, and Treatment—A Scoping Review

**DOI:** 10.1111/ger.70066

**Published:** 2026-05-15

**Authors:** Helena Dujic, Katrin Heck, Julia Schwärzler, Cristiane da Mata, Sayaka Tada, Falk Schwendicke

**Affiliations:** ^1^ Department of Conservative Dentistry, Periodontology and Digital Dentistry LMU University Hospital Munich Germany; ^2^ Department of Restorative Dentistry Cork Dental School and Hospital, University College Cork Cork Ireland; ^3^ Faculty of Dentistry National University of Singapore Singapore Singapore

**Keywords:** caries increment, caries occurrence, caries risk, elders, oral health‐related quality of life

## Abstract

**Background:**

Dental caries remains one of the most common chronic diseases affecting older adults worldwide. As edentulism decreases and more natural teeth are retained into old age, the number of susceptible tooth surfaces increases. Age‐related factors such as polypharmacy, cognitive decline, and reduced oral hygiene skills, together with systemic and social factors, increase the risk of caries. Although the literature is extensive, the evidence for diagnosis and management remains fragmented and lacking clinical integration.

**Objective:**

This scoping review aimed to assess and map current evidence on caries diagnostics and management in adults aged 60 years and older, focusing on diagnostic indices, associated factors, treatment strategies and outcomes, as well as oral health‐related quality of life (OHRQoL).

**Methods:**

A comprehensive search of MEDLINE, Embase, and the Cochrane Library identified 533 studies published between 2000 and 2025. Eligible studies included data on caries occurrence, incidence, increment, factors associated with caries, treatment, and OHRQoL in older adults.

**Results:**

Most studies focused on caries occurrence and associated factors, with DMFT being the most common index. Reported caries proportions varied widely across studies (0.1%–100%). Seventy‐five distinct variables were identified covering multiple domains, with older age, sex, and lower education being the most frequently cited. Only seven studies reported using risk assessment tools. Treatment strategies were less frequently addressed and often lacked feasibility or outcome metrics. OHRQoL was reported in 98 studies, mostly using GOHAI and OHIP.

**Conclusion:**

Current evidence focuses largely on epidemiological endpoints. Future studies should prioritize patient‐relevant outcomes and include frail and dependent populations.

**Trial Registration:**

PROSPERO: CRD420251004339

## 1 Introduction

Dental caries remains one of the most prevalent chronic conditions affecting older adults globally, with a high and often under‐recognised burden across populations [1, 2]. As edentulism has declined and natural tooth retention has improved, this shift has also increased the number of susceptible tooth surfaces in older age [1]. In response to demographic change, the World Health Organization has called for integrated, person‐centred healthcare strategies that explicitly include oral health as part of healthy ageing [3]. Similar to other chronic conditions such as diabetes or cardiovascular disease, caries is now widely recognised as a non‐communicable disease resulting from complex interactions between biological, behavioural, and social factors [4]. This reclassification supports its integration into broader health promotion and universal health coverage frameworks. Nevertheless, caries management in older adults remains poorly adapted to the chronic and complex needs of this population, particularly in frail or dependent individuals [2].

Ageing is accompanied by physiological and functional changes that elevate caries risk, including hyposalivation, polypharmacy, cognitive decline, and difficulties with oral hygiene [2, 5]. In this context, both coronal and root caries are clinically relevant. Specifically, root caries has been associated with gingival recession, attachment loss, and exposed dentine surfaces, which represent prevalent conditions in older adults [6, 7]. Since coronal and root lesions differ in aetiology, progression, and treatment needs, they are often analysed separately in epidemiological studies [2]. Root caries presents distinct management challenges, including differences in lesion accessibility, requirement for different restorative materials, and greater emphasis on non‐invasive strategies such as high‐concentration fluoride applications and daily lesion brushing compared to standard coronal caries management [2].

Beyond the clinical burden, caries significantly impairs oral health‐related quality of life (OHRQoL). Symptoms such as pain, chewing difficulties, and prosthetic complications interfere with eating, speaking, and social engagement, often leading to reduced autonomy and social withdrawal [8, 9]. Consequently, outcome assessments are increasingly expected to consider functional and psychosocial domains, rather than just clinical indicators [4].

To understand caries risk in older age, both individual and contextual factors or indicators should be considered. Typically, age, socioeconomic status, and institutionalisation, as well as diabetes, cognitive impairment, poor oral hygiene, xerostomia, and limited access to dental care are frequently reported factors associated with caries risk in older adults [2, 5]. However, inconsistent definitions and measurement tools compromise cross‐study comparability.

Expert consensus emphasises the importance of tailoring caries prevention and management to the individual's functional status and living environment. This involves integrating shared decision‐making, personalised treatment goals, and caregiver involvement where appropriate [2]. This review is intended to serve as a basis for the development of a framework for comprehensive and individualised diagnostics in older people, with the focus being on caries diagnostics and treatment planning in this group. Specifically, the objectives were to:

1. Map the range and consistency of diagnostic indices used to assess coronal and root caries occurrence, incidence, and increment in older adults, and identify gaps in standardisation.
2. Examine the range and heterogeneity of reported factors associated with caries in older adults and their assessment methods, and identify priorities for validated risk assessment tool development.
3. Characterise the scope of evidence on treatment strategies, treatment outcomes, and OHRQoL in this population, and identify gaps in outcome reporting and feasibility assessment.

## 2 Methods

This review was originally planned and registered as a systematic review in PROSPERO. During screening and data extraction, the large number of eligible studies, considerable methodological heterogeneity and variation in outcome measures, however, suggested that a meaningful quantitative synthesis would be unfeasible. To better reflect the broad, exploratory aims of the review and to enable a comprehensive review of the existing literature, the findings were adapted into a scoping review. The final review was conducted in accordance with the updated methodological guidelines of the Joanna Briggs Institute [10, 11] and reported according to the PRISMA‐ScR checklist [12].

### 2.1 Eligibility Criteria

Inclusion and exclusion criteria were defined a priori based on the review objectives to ensure transparency and consistency during study selection.

#### 2.1.1 Inclusion Criteria

Studies were included if they involved participants aged 60 years or older with data on coronal and/or root caries. Eligible studies reported on at least one of the following: caries occurrence, incidence or increment of coronal or root caries; caries risk assessment or factors associated with caries development or progression; treatment outcomes (e.g., restoration survival, tooth loss); or OHRQoL. Included study designs were cross‐sectional, cohort, or case–control studies, as well as systematic reviews and/or meta‐analyses. The inclusion time frame was from 2000 to 2025.

#### 2.1.2 Exclusion Criteria

Studies focused exclusively on populations younger than 60 years or reporting solely on primary teeth or non‐carious lesions were excluded. Studies involving highly specific clinical populations not generalisable to older adults (e.g., individuals undergoing cancer treatment or radiotherapy) were also excluded. Non‐original research (e.g., narrative, scoping, umbrella, or literature reviews; book chapters; commentaries) as well as case reports, case series, conference abstracts, and animal or in vitro studies were not considered for inclusion.

### 2.2 Search and Screening

A comprehensive search was conducted in MEDLINE (via Ovid and PubMed), Embase, and the Cochrane Library. Reference lists of included full texts and relevant systematic reviews were also screened manually to identify additional eligible studies. The search strategy was initially developed based on the PICOS framework, combining MeSH terms and free‐text keywords tailored to each database. It targeted studies involving older adults aged 60 years and above, including those living in the community and in institutional settings, and focused on the presence, incidence, increment, prevalence, risk factors, and treatment of coronal and/or root caries, as well as associated outcomes such as restoration survival, tooth loss, and OHRQoL. No restrictions were applied regarding care setting or geographic region. While no language limits were used in the initial search, studies without English abstracts were excluded if translation was not feasible. The search was conducted in early 2025.

All identified records were exported to Rayyan [13], where duplicates were removed. Screening was conducted in two phases by four independent reviewers. Titles and abstracts were first screened against the eligibility criteria. Full texts of potentially relevant articles were then imported into EndNote [14] and assessed independently by four reviewers. Discrepancies during either stage were resolved through discussion. Reasons for full‐text exclusion were documented in the data extraction sheet developed in EpiData Manager [15].

### 2.3 Data Extraction and Presentation

Included data covered study details (title, authors, publication year, journal and country of publication), study design, population characteristics, caries type, reported caries occurrence and incidence or increment data, factors associated with caries, treatment approaches, treatment outcomes, and OHRQoL. Data extraction was carried out independently by four reviewers using EpiData Entry Client [15]. The data was first extracted and visualised using Microsoft Excel [16]. Python (v3.12.7, http://www.python.org) was used to analyse the data and create the figures.

## 3 Results

In the following, the findings of the review are presented, including the number of studies identified and selected, key study characteristics, and an overview of findings related to caries occurrence, incidence, increment, risk assessment and factors, treatment approaches, treatment outcomes, and OHRQoL. The evidence is described narratively and grouped thematically according to the main concepts of interest.

### 3.1 Screening and Study Selection

**FIGURE 1:**
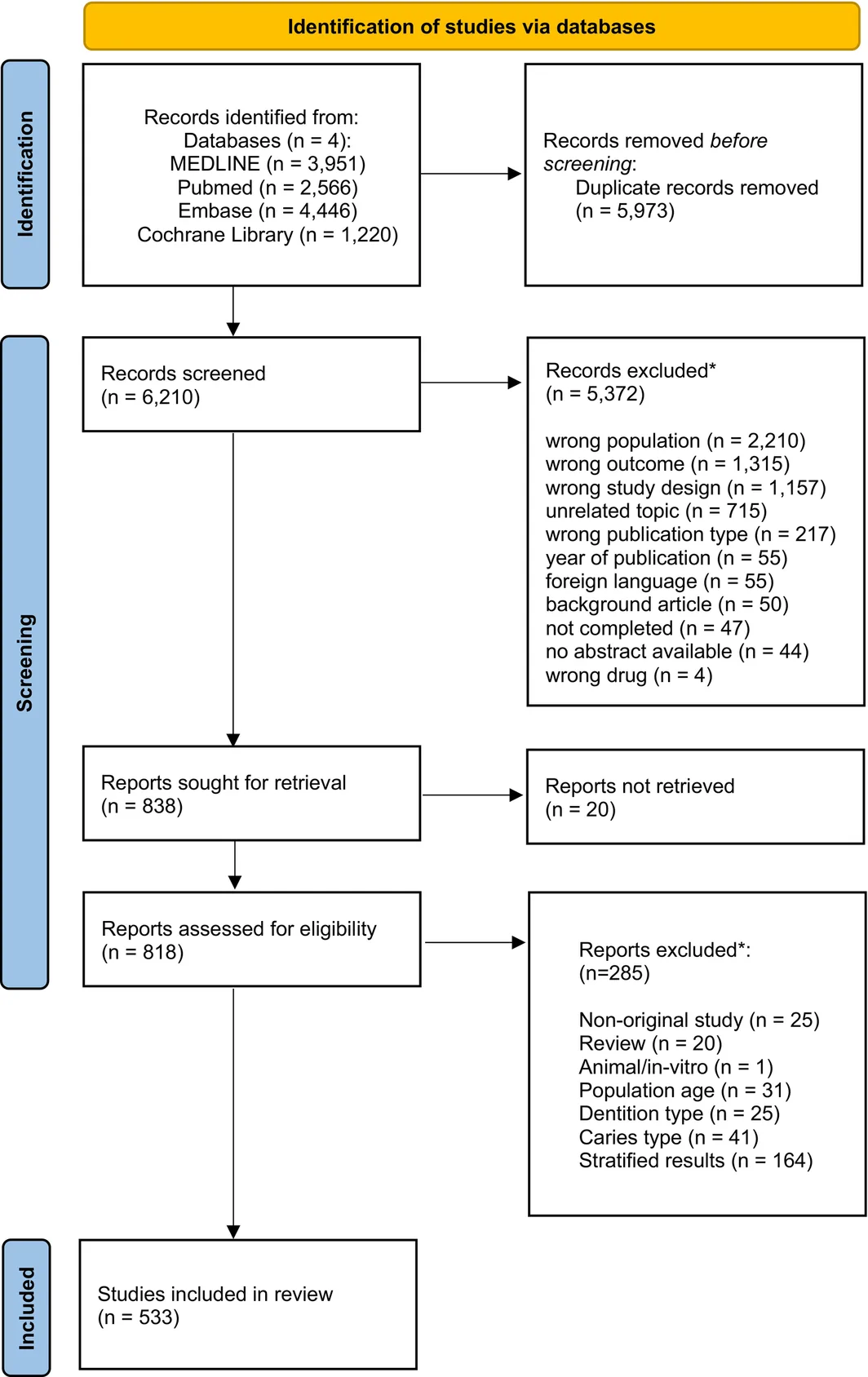
PRISMA flow diagram illustrating the selection process of studies included in the scoping review. Adapted from Page et al. 2021. *The number of studies excluded per individual exclusion reason exceeds the total number of excluded studies, since multiple exclusion criteria could be applied to a single study during title, abstract, and full‐text screening. [Colour figure can be viewed at wileyonlinelibrary.com]

The study selection process is illustrated in Figure 1. The search yielded a total of 6210 records after de‐duplication. Following title and abstract screening, 838 full texts were assessed for eligibility. Of these, 533 studies met the inclusion criteria and were included in the final synthesis. Reasons for exclusion during title and abstract screening are displayed in the PRISMA flow diagram (Figure  1). For full‐text screening, exclusion was based on predefined criteria including publication type, study design, population age, caries type, dentition type, and whether results were reported separately for individuals aged 60 years and older.

### 3.2 Study Characteristics

The 533 studies included in the review were published between 2000 and 2025. Most employed a cross‐sectional design, with 319 studies using cross‐sectional methods, followed by 90 population‐based surveys, 72 cohort studies, and 30 systematic reviews and/or meta‐analyses. An additional 22 studies were classified as “other” designs. Sample sizes ranged from 10 to 70,057 participants, and study settings included both community‐based and institutionalised older populations. While most studies included adults aged 60–79 years, several specifically focused on adults aged ≥ 85 years (*n* = 4) and ≥ 100 years (*n* = 1). This represents a 40+ year age span from the youngest (60 years) to the oldest (≥ 100 years). A list of the included studies is provided in Table S1.

### 3.3 Overview of Findings

The data synthesis revealed substantial variation in how caries occurrence, incidence, increment, associated factors, treatment, and outcomes were assessed across studies. Coronal caries was reported in 205 studies, root caries in 88 studies, and both coronal and root caries in 235 studies; five studies did not specify the caries type. Findings are presented narratively and grouped according to the main review objectives: diagnostic indices for caries assessment, risk assessment and factors, treatment approach and outcomes including OHRQoL.

#### 3.3.1 Indices Used to Assess Caries Occurrence, Incidence and Increment

In cross‐sectional studies of populations aged 60 years and older, reported caries proportions ranged widely: 0.2% to 100% for coronal caries and 0.1% to 95.3% for root caries. The most frequently used index for assessing caries lesions was the Decayed, Missing, and Filled Teeth (DMFT) index (*n* = 270; 62% of studies), followed by the Root Caries Index (RCI) (*n* = 26; 6% of studies) and the Decayed, Missing, and Filled Surfaces (DMFS) Index (*n* = 26; 6% of studies). Additional or modified indices, including International Caries Detection and Assessment System (ICDAS), Decayed Teeth (DT), Decayed Surfaces (DS), Decayed and Filled Teeth (DFT), Decayed and Filled Surfaces (DFS), Decayed and Filled Root Surfaces (RDFS), modified DFT index for root caries (RDFT), and DT index modified for root caries (RDT), were used in 110 studies (25%). Variations in application were observed across studies using the same index, including differences in diagnostic thresholds (e.g., cavitated vs. non‐cavitated lesions), criteria for surface involvement, and whether missing teeth were included in calculations.

**FIGURE 2:**
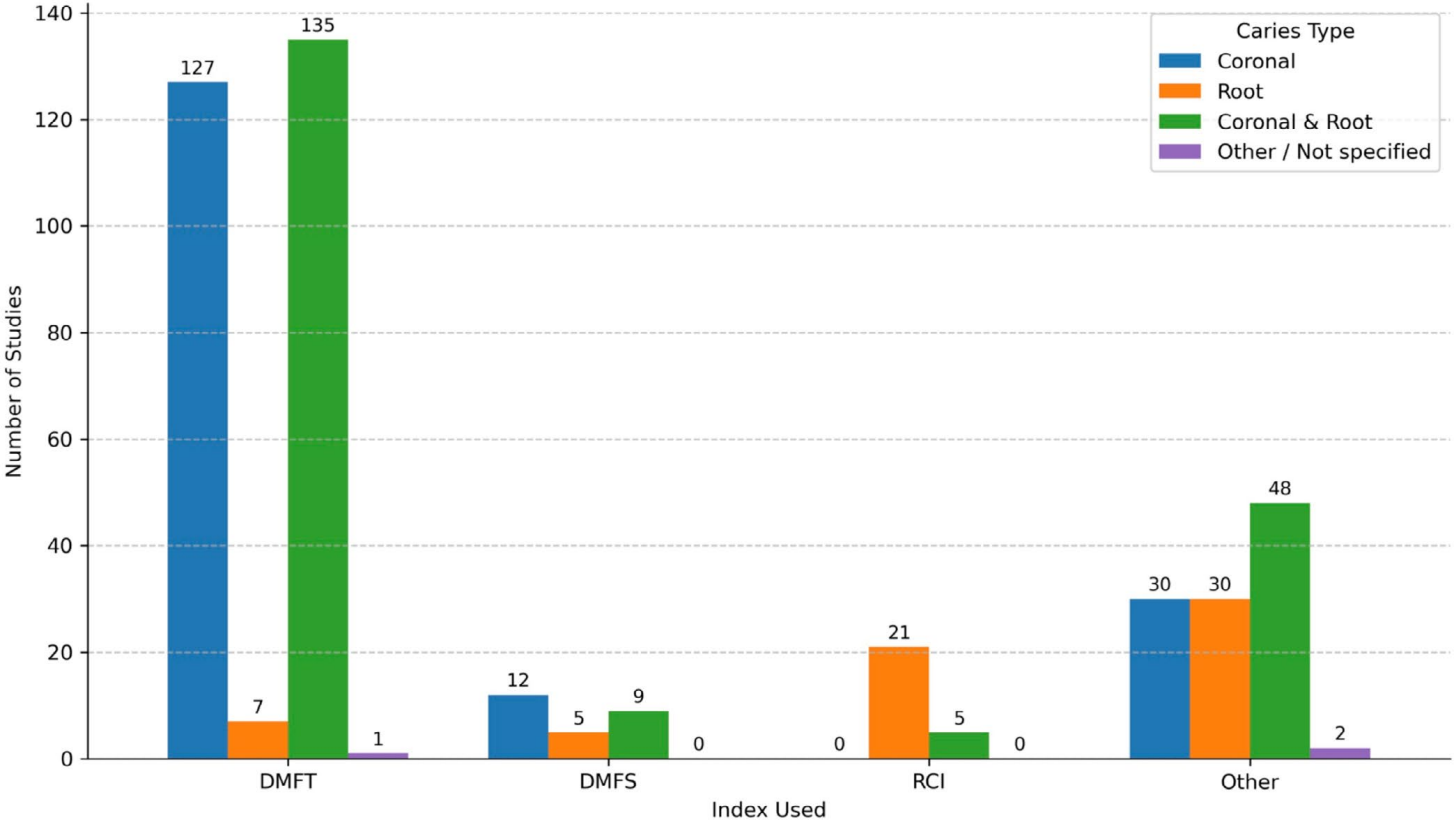
Diagnostic indices used for the assessment of caries occurrence in older adults (*n* = 432 studies). The number of studies using each index (DMFT, DMFS, RCI, and others) is stratified by caries type (coronal, root, both coronal and root, or unspecified). Values above each bar indicate the number of studies reporting each index by caries type. [Colour figure can be viewed at wileyonlinelibrary.com]

**FIGURE 3:**
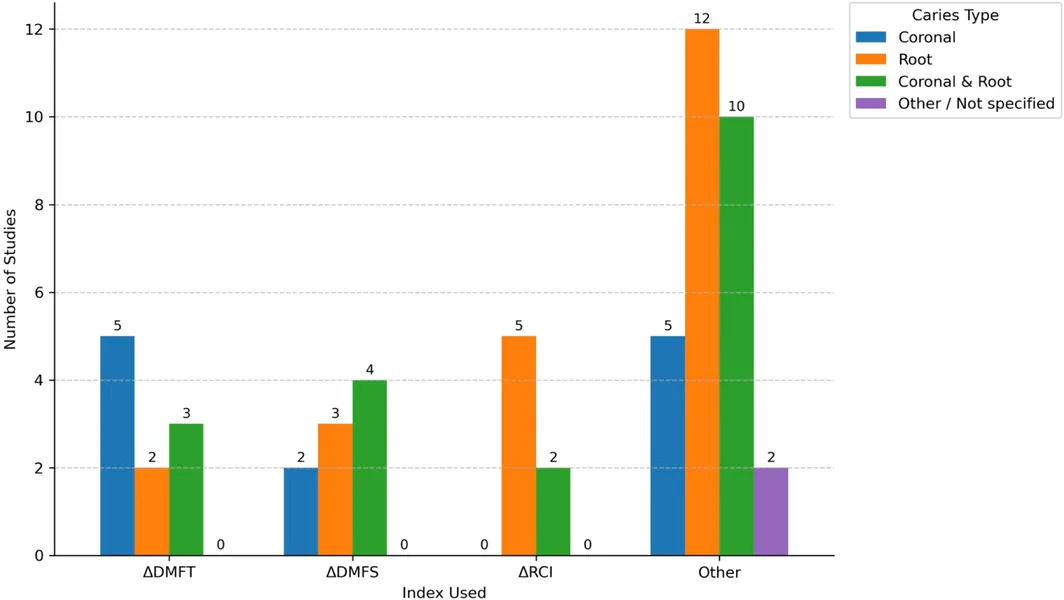
Diagnostic indices used for the assessment of caries increment in older adults (*n* = 55 studies). The reported indices (ΔDMFT, ΔDMFS, ΔRCI, and other indices) are stratified by caries type (coronal, root, both coronal and root, or unspecified). Counts above each bar indicate the number of studies using each index by caries type. [Colour figure can be viewed at wileyonlinelibrary.com]

Caries incidence or increment was reported in 55 longitudinal studies, using ΔDMFT (*n* = 10; 18%), ΔDMFS (*n* = 9; 16%), and ΔRCI (*n* = 7; 13%), while 29 studies (53%) applied less standardised or study‐specific indices such as Decayed Surfaces (DS), Decayed Root Surfaces (DRS), Root Caries Assessment Index (RCAI), Decayed and Filled Surfaces (DFS%), Decayed and Filled Coronal Surfaces (DFCS%), and Decayed and Filled Root Surfaces (DFRS%). Among the seven studies using ΔRCI, three employed surface‐by‐surface methodology with reversal correction [17, 18, 19], three did not report or explicitly did not correct for reversals [20, 21, 22], and one was a modelling study using secondary data [23]. Methodological details for increment calculation were not consistently reported across studies using other indices. Figures 2 and 3 summarise the distribution of indices used to assess caries occurrence and increment.

#### 3.3.2 Caries Risk Assessment and Associated Factors

**FIGURE 4:**
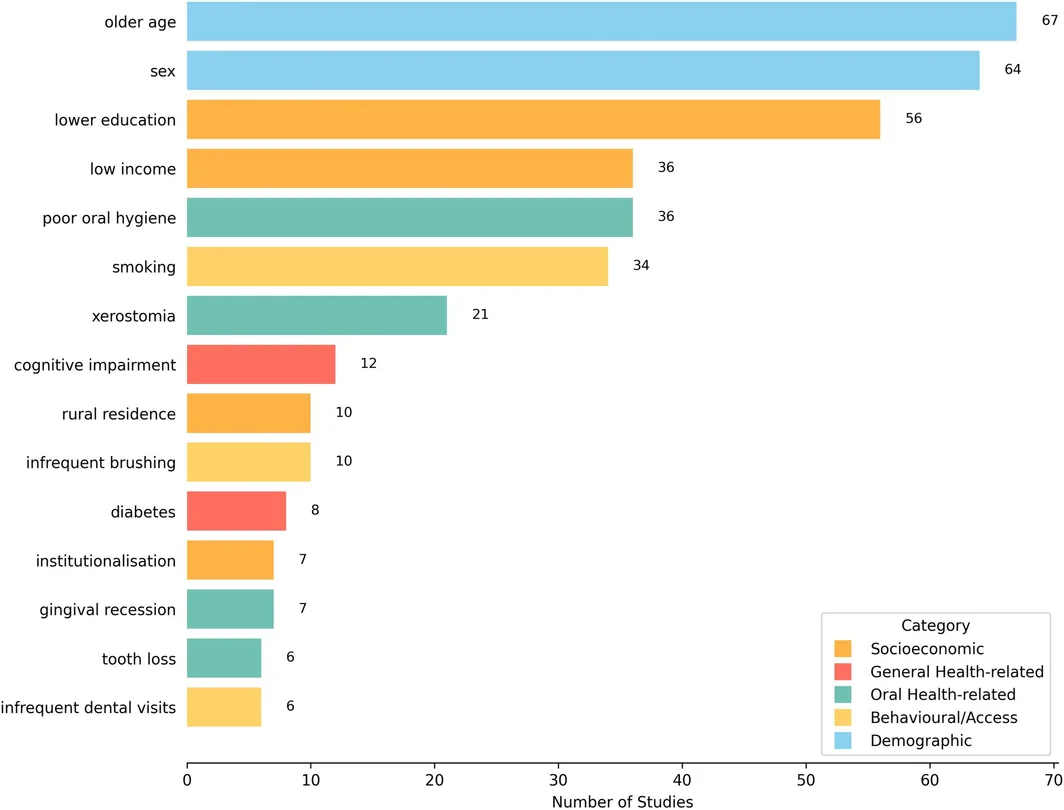
Reported factors associated with caries in older adults, categorised by conceptual domain. The most frequently reported factors for caries are grouped by the following categories: Demographic, socioeconomic, behavioural/access‐related, oral health‐related, and general health‐related. Each bar indicates the total number of studies a risk factor was reported across included studies (*n* = 337). [Colour figure can be viewed at wileyonlinelibrary.com]

Factors associated with caries development and progression were reported across a wide range of studies. In total, 75 distinct terms were identified, with variation in terminology, definitions, and reporting formats. Figure 4 displays the most frequently reported factors, grouped by conceptual domain. Frequently cited factors included older age (*n* = 67), sex (*n* = 64), lower educational level (*n* = 56), poor oral hygiene (*n* = 36), and smoking (*n* = 34). Additional factors such as lower income, xerostomia, cognitive impairment, institutionalisation, and diabetes were also frequently reported. However, for sex, associations were inconsistent across studies, with no clear predominant direction identified.

In addition to individual associated factors, seven studies described the use of structured risk assessment tools to evaluate caries susceptibility in older adults. Reported tools included the Cariogram, Caries Management by Risk Assessment (CAMBRA), the Clinical Index of Oral Disorder in Elders (CODE) odontogram, ICDAS‐II, a personalised nomogram, and administrative classification systems such as dental procedure codes. These models varied in their complexity and the range of variables assessed. Input parameters commonly included clinical indicators such as the number of remaining teeth, plaque scores, DMFT, or RCI; salivary factors such as flow rate and buffering capacity; and broader determinants including general health status, cognitive function, and capacity for oral self‐care. Some tools incorporated sociodemographic and behavioural variables as part of a multidimensional risk profile. Two tools underwent formal validation procedures for use in older adults: Cariogram and the personalised nomogram. An overview is provided in Table 1.

**TABLE 1:** Overview of structured caries assessment tools identified across included studies, ranging from multifactorial risk prediction models to diagnostic classification systems and clinical documentation tools.

Risk assessment tool	Variables assessed	Validation in older adults
Cariogram [21, 24]	Medical history, fluoride exposure, diet, saliva (flow rate, buffer capacity, bacterial counts) DMFT, plaque scores, oral hygiene	Validated for root caries prediction in 2‐year cohort; stratified older adults into risk groups
CAMBRA [25]	Caries indicators, risk factors, protective factors	Applied descriptively; not formally validated in older adult populations
Nomogram (personalised) [26]	Oral health, frailty (FRIED), cognition (MMSE), function (Barthel Index), xerostomia (SXI), QoL (OHIP‐14), demographics	Internal validation
CODE odontogram [27]	Caries status, demographics, medical comorbidities, mobility, oral hygiene	Limited, primarily documentation system; validated in residential‐care
ICDAS II [28]	Coronal and root caries severity, converted to DMFT/DFRS/RCI, demographics, functional impairment, oral hygiene assistance needs	Not validated as a predictive risk assessment tool
Current Dental Terminology (CDT) Codes [29]	Treatment history, demographics, comorbidity indices, medications, dental behaviours, restorations	Administrative classification system; applied descriptively to identify high‐risk patients

#### 3.3.3 Treatment Approach and Outcomes

Treatment strategies were reported with varying frequency across the included studies. Preventive measures were described in 67 studies, followed by restorative treatments (*n* = 28), supportive therapies (*n* = 22), and multidisciplinary care approaches (*n* = 20). Minimally invasive (*n* = 10), micro‐invasive (*n* = 9), and surgical interventions, such as tooth extractions (*n* = 9), were less commonly addressed. Among studies with known residential status, institutionalised populations reported treatment strategies more frequently (22.2%; 26/117 studies) than community‐dwelling populations (8.9%; 25/281 studies), with higher reporting across restorative treatment (11.1% compared to 4.6%), supportive therapy (11.1% compared to 2.8%), and surgical interventions (5.1% compared to 1.1%).

Outcome reporting was substantially less frequent than treatment description. Among the 165 studies describing treatment strategies, specific outcomes were reported in fewer than one‐third. Restoration survival (*n* = 16), tooth loss (*n* = 13), and retreatment rates (*n* = 8) were addressed more selectively. Very few studies reported on post‐treatment complications (*n* = 2), adverse events (*n* = 1), or cost‐effectiveness (*n* = 6). Standardised outcome measures were not consistently applied, and definitions of treated teeth vary across studies, precluding quantitative synthesis.

#### 3.3.4 Oral Health‐Related Quality of Life

OHRQoL was reported in 98 of the included studies (18%), using a combination of validated and self‐reported instruments. Eighty‐four studies (86%) used at least one validated tool, most commonly the Geriatric Oral Health Assessment Index (GOHAI, *n* = 43) and the Oral Health Impact Profile (OHIP, *n* = 35), including OHIP‐14 and OHIP‐49. Other validated tools included the Oral Impacts on Daily Performance (OIDP, *n* = 8), the World Health Organization Quality of Life‐BREF (WHOQOL‐BREF, *n* = 2), and the Oral Health Impact on Daily Living (OHIDL, *n* = 1). Twenty studies (20%) used self‐reported or non‐validated instruments, some alongside validated tools. The combined use of multiple tools was common in five studies.

## 4 Discussion

This scoping review synthesized evidence from 533 studies on caries in adults aged 60 years and older published over a 25‐year period. Most studies employed cross‐sectional designs and were conducted in community‐dwelling populations. A total of 123 studies included institutionalized or hospitalized older adults. The findings highlight considerable variation in focus, methods, and outcomes across the existing literature. While most studies reported on caries occurrence or associated factors, fewer addressed incidence, risk assessment tools, treatment strategies, treatment outcomes, or outcomes related to OHRQoL.

However, several limitations should be acknowledged. First, consistent with scoping review methodology, no risk of bias assessment was conducted, and findings were not pooled quantitatively. Second, considerable heterogeneity in study designs, outcome definitions, and measurement tools limited comparability and precluded the aggregation of data across studies. Furthermore, older adults remain relatively underrepresented considering their elevated caries risk and dental treatment needs [18, 28, 30, 31, 32, 33, 34]. This limits the generalisability of findings to frail older adults in long‐term care or hospital settings, despite their importance as a key segment of the ageing population. Lastly, variations in terminology and reporting across behavioural, systemic, and treatment‐related domains limited the interpretability of some findings.

The evidence base reflects a wide variation in diagnostic indices, associated factor definitions, and outcome measures, underscoring both the complexity of caries in older age and the challenges of synthesising findings across contexts. The following sections discuss key findings in relation to the three core review questions: diagnostic indices, caries risk assessment and factors, and treatment and OHRQoL. Diagnostic indices used to assess caries occurrence, incidence and increment varied considerably across the included studies. The DMFT was by far the most commonly used and reported index [28, 35, 36, 37, 38, 39, 40, 41, 42, 43], whereas RCI and DMFS were reported less frequently [18, 44, 45, 46, 47, 48, 49, 50]. Only a small number of studies applied more detailed diagnostic systems such as the ICDAS or extended indices for root caries [6, 50]. This widespread reliance on the DMFT index underscores its established role in population‐level caries surveillance. However, its limitations, such as the inability to differentiate between active and arrested lesions or to capture current disease activity, particularly on root surfaces, restrict its usefulness for nuanced risk assessment or clinical decision‐making. Furthermore, inconsistencies in the use of these indices, such as different diagnostic thresholds, criteria for surface involvement, or inclusion of missing teeth, limit comparability between studies. This highlights the need for greater standardisation in caries diagnostics for older age groups. In addition, the reported proportions for coronal and root caries reflect considerable heterogeneity in study populations, methods and diagnostic criteria.

Across included studies, reported caries occurrence ranged from 0.2% to 100% for coronal caries and 0.1% to 95.3% for root caries. These figures are not directly comparable, as they reflect fundamental differences in what was measured—from untreated lesions to cumulative caries burden as captured by DMFT > 0. Additionally, some intervention trials required presence of root caries as an inclusion criterion [51, 52]. Notably, our analysis did not systematically distinguish between representative population‐based samples and convenience samples (e.g., clinical populations), which may partially explain the wide variation and limit generalizability of these estimates.

Qualitative analyses across settings suggested differences in caries burden and assessment approaches. Studies in institutionalised populations more frequently reported higher proportions and greater oral hygiene challenges compared to community‐dwelling populations. DMFT remained the predominant index across all settings, while root‐specific indices were more commonly applied in studies of older age groups where root exposure was more prevalent. However, the lack of standardised sampling methods, diagnostic criteria, and systematic coding of sample representatives precluded quantitative comparison across settings. The findings suggest that no single index is universally appropriate for older adults, but rather that index selection should be tailored to the specific assessment purpose and population context. For population‐level surveillance and comparability with existing data, DMFT remains valuable due to its widespread use and established normative data. However, for clinical risk assessment and treatment planning in older adults, root‐specific indices such as RCI provide more relevant information given the high prevalence of gingival recession and root exposure in this age group. The limited use of more detailed systems such as ICDAS (reported in only 9 studies) represents a missed opportunity for distinguishing between active and arrested lesions, which is particularly important in older adults where lesion activity status informs treatment decisions. Future research should prioritise indices that capture root caries, lesion activity, and treatment complexity relevant to older adults' unique clinical presentations.

Furthermore, longitudinal studies reported caries increment using various indices, most commonly ΔDMFT, ΔDMFS, and ΔRCI. However, methodological approaches varied considerably in their rigour. Proper determination of caries increment requires surface‐by‐surface comparisons between baseline and follow‐up examinations with correction for reversals, rather than simple subtraction of baseline scores from follow‐up scores. Among studies using ΔRCI, only three of seven employed this rigorous methodology [17, 18, 19], with the remainder either not reporting reversal correction or explicitly stating that no correction was made. This methodological inconsistency limits the comparability and reliability of reported increment data across studies. Additionally, many studies reported increment (mean change in scores) rather than incidence (proportion developing ≥ 1 new lesion), which represents fundamentally different epidemiological measures.

As noted in the results, substantial age heterogeneity was also observed, reflecting the age difference between 60‐year‐olds and centenarians, on which some of the studies also focused [53]. This 40+ year age range has important implications for interpreting caries occurrence estimates and treatment approaches, as the oral health needs, functional capacity and biological characteristics of adults in their early sixties differ markedly from those of centenarians. In view of this, future studies should further emphasize the age differences in the population over 60 years in order to take into account the possible changes that may affect not only oral health but also quality of life.

This review identified 75 distinct variables associated with caries development and progression. The range of factors examined, such as sociodemographic determinants [35, 54, 55], behavioural factors [56, 57, 58], and geriatric‐specific indicators [5, 59, 60, 61], reflect the multifactorial nature of caries susceptibility in this population. The most commonly reported factors are shown in Figure 4. However, considerable variation existed in how these variables were defined and measured across studies, combined with differences in study populations, outcome measures, and statistical approaches, which complicates cross‐study comparability and limits the potential for pooled estimates or meta‐analytical synthesis.

Beyond reporting individual associated factors, seven studies employed structured risk assessment tools (Table 1), though adoption of such tools remains limited. The tools varied considerably in their validation status and complexity: only two, the Cariogram and a personalised nomogram [21, 24, 26], underwent formal validation in older adult populations. Other tools, such as CAMBRA and the CODE odontogram, were applied descriptively or primarily for documentation purposes [25, 27]. The limited adoption and validation of multifactorial risk assessment instruments represent a significant gap, particularly given the complex interplay of medical, functional, cognitive, and social factors affecting caries risk in older adults. Given the increasing need for individualised prevention and care planning in ageing populations, the development and validation of standardised, evidence‐based caries risk assessment models adjusted to older adults should be prioritised in future research and practice. Based on this review's findings, such tools should incorporate not only traditional oral health indicators (root and coronal caries status, saliva function, oral hygiene capacity) but also geriatric‐specific domains including functional status, cognitive function, frailty level, polypharmacy, and care setting. The tool developed by Huang et al. provides a useful framework by integrating the Fried Frailty Phenotype, Mini‐Mental State Examination, and Barthel index alongside clinical oral health measures [26]. However, further validation across diverse populations and assessment of clinical utility and feasibility in different care settings remain essential.

When considering treatment approaches, feasibility, and outcomes, relatively few studies have addressed these aspects in depth for caries in older adults. Preventive strategies were discussed primarily in the context of coronal caries [62, 63]. Furthermore, restorative interventions, including those targeting both coronal and root caries, were described across several studies [64, 65, 66]. Supportive elements, such as prosthetic management and assistance with oral hygiene, were also included in a few studies [62, 67]. Multidisciplinary approaches, although reported infrequently, appeared in the context of complex care needs [62, 68]. Despite the relevance of these interventions for older adults with multimorbidity, frailty, or cognitive impairment, treatment feasibility was rarely addressed explicitly. These findings align with recent reviews emphasising that the evidence base for managing root caries in older adults remains limited and inconclusive, underlining the need for further research [7, 69]. Treatment reporting appeared more frequent in studies of institutionalised populations compared to community‐dwelling populations, particularly for restorative treatment, supportive therapy, and surgical interventions. However, these differences should be interpreted with caution, as included studies varied in whether they reported treatments delivered, treatment needs assessed, or recommendations made.

Outcome reporting across the included studies was similarly inconsistent. Only a subset reported on specific outcomes such as restoration survival [70, 71], tooth loss [17, 51, 72, 73], or retreatment rates [66, 74]. Very few studies addressed cost‐effectiveness [23, 75] or post‐treatment complications [76, 77]. This lack of systematic outcome reporting limits the ability to evaluate the broader implications of caries interventions in this age group. Moreover, the lack of data on effectiveness, safety, and economic impact poses significant challenges for clinical decision‐making and cost–benefit assessments in geriatric dental care.

In contrast to treatment outcomes, a comparatively larger number of studies (*n* = 98) reported on OHRQoL, often using validated instruments. The most commonly used tools were the GOHAI and OHIP [9, 78, 79, 80]. These instruments helped capture the functional and psychosocial burden of caries including impacts on eating, speech, pain, and social interaction. Several studies also assessed OHRQoL in relation to clinical factors such as edentulism, root caries, denture hygiene, and perceived treatment needs. In addition, some studies combined OHRQoL tools with broader health or psychosocial measures [9, 81, 82, 83]. However, only a limited number of studies explicitly examined the direct impact of caries on OHRQoL, typically linking untreated or advanced carious lesions to lower quality of life scores [84, 85]. Given the variety of instruments used, the different assessment and the use of non‐validated or self‐developed instruments in some cases, the comparability of the studies and the synthesis of the findings remain limited.

Despite the limitations, this review highlights several implications for research and dental practice. The standardization of diagnostic criteria and caries indices is necessary in order to improve comparability and clinical benefit, especially for root surfaces. Structured and validated risk assessment tools adapted for older adults should be more widely developed, applied, and validated, particularly for use in diverse care settings. In addition, treatment feasibility and outcome reporting require greater emphasis, including aspects such as cost, adverse events, and follow‐up, which are particularly relevant in assessing the long‐term value and safety of caries interventions in older populations. Finally, future studies should systematically include institutionalized and care‐dependent populations and report findings by age group and functional status to support more targeted caries management strategies.

## 5 Conclusions

This scoping review of 533 studies reveals important progress in documenting caries burden in older adults, alongside critical opportunities to strengthen the evidence base for clinical practice. While caries occurrence and associated factors have been extensively examined, three key areas require further development to support evidence‐informed care. First, diagnostic approaches would benefit from greater standardisation and adaptation to older adults' needs, particularly regarding root caries assessment and differentiation between active and arrested lesions. Second, structured risk assessment tools require broader validation in older adult populations as well as incorporation of geriatric‐specific factors such as frailty, cognitive function, and functional status. Third, treatment research would be strengthened by more systematic evaluation of feasibility, safety, and patient‐centred outcomes, with greater inclusion of institutionalised and cognitively impaired populations who face elevated caries risk yet remain underrepresented in current research. Addressing these gaps will require development of standardised diagnostic and risk assessment tools, systematic reporting of treatment outcomes across diverse care settings, and research designs that ensure adequate representation of care‐dependent populations. These advances would support the translation of epidemiological knowledge into practical strategies for prevention and management of caries in ageing populations.

## Author Contributions

All authors contributed meaningfully to this review and meet the criteria for authorship. Falk Schwendicke, Helena Dujic, and Katrin Heck conceptualised and designed the study. Helena Dujic was responsible for data collection. Title and abstract screening were performed by Helena Dujic, Katrin Heck, Cristiane da Mata, and Sayaka Tada; full text screening was conducted by Katrin Heck, Cristiane da Mata, Julia Schwärzler, and Helena Dujic. Data analysis and interpretation were carried out by Falk Schwendicke and Helena Dujic. Falk Schwendicke provided critical revision of the manuscript, with input and review from all other authors. All authors have read and approved the final version of the manuscript.

## Funding

The authors have nothing to report.

## Ethics Statement

This study is a scoping review based on previously published literature. It did not involve human participants, interventions, or the collection of personal data. Therefore, ethical approval and informed consent were not required.

## Conflicts of Interest

The authors declare no conflicts of interest.

## Supporting information


**Table S1:** Summary of included studies in the scoping review (*N* = 533).

## Data Availability

The data that support the findings of this study are available from the corresponding author upon reasonable request.
