# Supplementary material for: Coronal and Root Caries in Older Adults: Associated Factors, Quality of Life Impact, and Treatment—A Scoping Review
Source: Gerodontology. 2026 May 15;43(3):306–17. doi: 10.1111/ger.70066 (PMC13498928; doi:10.1111/ger.70066)
Supplement: Supplementary file 1 [file GER-43-306-s001.docx]

**Supplementary material**

Table S1	Summary of included studies in the scoping review (N = 533).

| **First Author & year** | **Title** | **Journal** | **Study design** |
| --- | --- | --- | --- |
| AlBaker 2017 | Oral Health Status and Oral Health-related Quality of Life among Hospitalized and Nonhospitalized Geriatric Patients | The Journal of Contemporary Dental Practice | Cross-sectional study |
| Adegbembo 2000 | National pathfinder survey of dental caries prevalence and treatment needs in The Gambia | Journal of the South African Dental Association | Epidemiological study |
| Ahluwalia 2010 | Oral disease experience of older adults seeking oral health services | Gerodontology | Cross-sectional study |
| Delbari 2024 | Evaluation of oral health status in the population above 50: evidence from the ardakan cohort study on aging (ACSA) | BMC Oral health | Cross-sectional study |
| Bianco 2021 | Oral Health Status and the Impact on Oral Health-Related Quality of Life among the Institutionalized Elderly Population:A Cross-Sectional Study in an Area of Southern Italy | International Journal of Environmental Research and Public Health | Cross-sectional study |
| Cruz Gonzalez 2016 | Clinical outcome of root caries restorations using ART and rotary techniques in institutionalized elders | Brazilian Oral Research | Cohort study |
| Chan 2021 | A Systematic Review on Caries Status of Older Adults | International Journal of Environmental Research and Public Health | Systematic review and/or meta analysis |
| Chan 2022 | Clinical evidence for professionally applied fluoride therapy to prevent and arrest dental caries in older adults: A systematic review | Journal of Dentistry | Systematic review and/or meta analysis |
| Barbe 2019 | Efficacy of regular professional brushing by a dental nurse for 3 months in nursing home residents‚ÄîA randomized, controlled clinical trial | International Journal of Dental Hygiene | Cohort study |
| Armfield 2009 | Dental fear and adult oral health in Australia | Community Dentistry and Oral Epidemiology | Cross-sectional study |
| Arrivé 2012 | Oral health condition of French elderly and risk of dementia: a longitudinal cohort study | Community dentistry and oral epidemiology | Epidemiological study |
| Balasubramaniam 2021 | Determining Association Between Cognitive Function and Oral Health Status among Rural Community Dwelling Geriatrics | Journal of International Society of Preventive & Community Dentistry | Cross-sectional study |
| Avlund 2003 | Social relations as determinants of oral health among persons over the age of 80 years | Community Dentistry and Oral Epidemiology | Cross-sectional study |
| Beltran-Aguilar 2005 | Surveillance for Dental Caries, Dental Sealants, Tooth Retention, Edentulism, and Enamel Fluorosis United States, 1988--1994 and 1999--2002 | Morbidity and mortality weekly report. Surveillance summaries | Epidemiological study |
| Bidinotto 2018 | Four-year incidence rate and predictors of root caries among community-dwelling south Brazilian older adults | Community dentistry and oral epidemiology | Epidemiological study |
| da Mata 2019 | An RCT of atraumatic restorative treatment for older adults: 5 year results | Journal of Dentistry | RCT |
| Croonquist 2020 | Effects of domiciliary professional oral care for care-dependent elderly in nursing homes - oral hygiene, gingival bleeding, root caries and nursing staff's oral health knowledge and attitudes | Clinical Interventions in Aging | Cohort study |
| Celeste 2010 | Why is there heterogeneity in the effect of dental checkups? Assessing cohort effect | Community dentistry and oral epidemiology | Epidemiological study |
| Chalmers 2005 | Caries incidence and increments in Adelaide nursing home residents | Spec Care Dentist | Cohort study |
| Chi 2013 | Correlates of root caries experience in middle-aged and older adults in the Northwest Practice-based REsearch Collaborative in Evidence-based DENTistry research network | Journal of the American Dental Association | Cross-sectional study |
| Choi 2011 | Does regional socioeconomic context affect the dental caries experience? A multilevel study of Korean adults | European journal of oral sciences | Cross-sectional study |
| Chou 2023 | Screening, Referral, Behavioral Counseling, and Preventive Interventions for Oral Health in Adults: A Systematic Review for the US Preventive Services Task Force | JAMA | Systematic review and/or meta analysis |
| Christensen 2012 | A cross-sectional study of oral health and oral health-related quality of life among frail elderly persons on admission to a special oral health care programme in Copenhagen City, Denmark | Gerodontology | Cross-sectional study |
| Besimo 2015 | Orale Gesundheit von Menschen mit Demenz | Schweiz Z Ganzheitsmed | Epidemiological study |
| Andersen 2025 | Prediction of oral diseases in care dependent older people | BMC Oral Health | Cross-sectional study |
| Chu 2015 | Oral Health Status of Elderly Chinese with dementia in Hong Kong | Oral health and Preventive Dentistry | Cross-sectional study |
| Coccia 2015 | Oral health conditions of the elderly in Ancona: a population-based study | Minerva stomatologica | Epidemiological study |
| da Mata 2015 | Two-year survival of ART restorations placed in elderly patients: A randomised controlled | Journal of Dentistry | RCT |
| da Mata 2015 | Subjective impact of minimally invasive dentistry in the oral health of older patients | Clinical oral investigations | Cohort study |
| Brennan 2000 | Caries experience among publicly-funded dental patients in Australia,1995-96: Type of care and geographic location | Australian Dental Journal | Cross-sectional study |
| Brennan 2004 | Changes in caries experience among Australian public dental patients between 1995/96 and 2001/02 | Australian and New Zealand Journal of Public Health | Cross-sectional study |
| Brennan 2011 | Caries experience of adults attending private and public dental clinics in Australia | Journal of Public Health Dentistry | Cross-sectional study |
| de Andrade 2013 | Relationship Between Oral Health and Frailty in Community-Dwelling Elderly Individuals in Brazil | Journal of the American Geriatrics Society | Cross-sectional study |
| De Marchi 2015 | Four-year incidence and predictors of coronal caries in south Brazilian elderly | Community dentistry and oral epidemiology | Cohort study |
| Ceena 2022 | Oral health status among the geriatric population - A cross sectional study | Journal of Gerontology and Geriatrics | Cross-sectional study |
| Agustina 2022 | Oral Conditions as Risk Factors for Low Oral Health-Related Quality of Life Among the Elderly Population in Yogyakarta, Indonesia | Europena Journal of Dentistry | Cross-sectional study |
| Brennan 2007 | Oral health of Indigenous adult public dental patients in Australia | Australian Dental Journal | Cross-sectional study |
| Du 2009 | Root caries patterns and risk factors of middle-aged and elderly people in China | Community Dentistry and Oral Epidemiology | Cross-sectional study |
| Dye 2012 | Selected oral health indicators in the United States, 2005-2008 | NCHS data brief | Epidemiological study |
| Dye 2007 | Trends in oral health status: United States, 1988-1994 and 1999-2004 | Vital and health statistics. Series 11, Data from the National Health Survey | Epidemiological study |
| Dye 2015 | Dental caries and tooth loss in adults in the United States, 2011-2012 | NCHS data brief | Cross-sectional study |
| Bozdemir 2016 | General health and oral health status in elderly dental patients in Isparta, Turkey | Eastern Mediterranean Health Journal | Cross-sectional study |
| Edman 2016 | Prevalence of dental caries and influencing factors, time trends over a 30-year period in an adult population. Epidemiological studies between 1983 and 2013 in the county of Dalarna, Sweden | Acta odontologica Scandinavica | Epidemiological study |
| Edman 2018 | Attitudes to dental care, Sweden 2003-2013, and clinical correlates of oral health-related quality of life in 2013 | International journal of dental hygiene | Cross-sectional study |
| Edman 2012 | Comparison of oral status in an adult population 35-75 year of age in the county of Dalarna, Sweden in 1983 and 2008 | Swedish dental journal | Cross-sectional study |
| Edman 2021 | Caries disease among an elderly population-A 10-year longitudinal study | International journal of dental hygiene | Cohort study |
| Ekanayake 2004 | The association between clinical oral health status and oral impacts experienced by older individuals in Sri Lanka | Journal of oral rehabilitation | Cross-sectional study |
| El Gedaily 2024 | Oral health, treatment burden and demographics of elders with care-resistant behavior: A cross-sectional study | Special care in dentistry | Cross-sectional study |
| El Osta 2018 | Social inequalities in oral health in a group of older people in a Middle Eastern country: a cross-sectional survey | Aging clinical and experimental research | Cross-sectional study |
| El Osta 2012 | Comparison of the OHIP-14 and GOHAI as measures of oral health among elderly in Lebanon | Health and quality of life outcomes | Cross-sectional study |
| Ellefsen 2008 | Caries prevalence in older persons with and without dementia | Journal of the American Geriatrics Society | Cross-sectional study |
| Ellefsen 2009 | Assessing caries increments in elderly patients with and without dementia: a one-year follow-up study | Journal of the American Dental Association | Cohort study |
| Esmeriz 2012 | Self-perception of oral health in non-institutionalised elderly of Piracicaba city, Brazil | Gerodontology | Cross-sectional study |
| Eustaquio 2010 | Oral health survey of the adult population of the Valencia region (Spain) | Medicina oral, patologia oral y cirugia bucal | Cross-sectional study |
| Fagundes 2022 | Factors associated with self-perceived oral health in different age groups | Community dentistry and oral epidemiology | Cross-sectional study |
| Fairhall 2009 | Home or away? Differences between home- and clinic-based dental examinations for older people | Gerodontology | Cohort study |
| Farias 2020 | Farias, Ilky Pollansky Silva E.Sousa, Simone Alves deAlmeida, Leopoldina de Fatima Dantas deSantiago, Bianca MarquesPereira, Antonio CarlosCavalcanti, Yuri Wanderley | Ciencia & saude coletiva | Systematic review and/or meta analysis |
| Farsangi 2022 | Oral Health Status and Related Factors among Adult Patients Referred to Public Health Centers (Kerman-Iran) | Journal of Kerman University of Medical Sciences | Cross-sectional study |
| Badr 2020 | Inequalities in Untreated Root Caries and Affordability of Dental Services among Older American Adults | International Journal of Environmental Research and Public Health | Epidemiological study |
| Ferracane 2011 | Use of caries prevention services in the Northwest PRECEDENT dental network | Community Dentistry and oral epidemiology | Cross-sectional study |
| Ferreira 2009 | Oral health among institutionalized elderly in Belo Horizonte, Minas Gerais State, Brazil | Cadernos de saude publica | Cross-sectional study |
| Ferro 2008 | Oral health problems and needs in nursing home residents in Northern Italy | Community Dental Health | Cross-sectional study |
| Almomani 2017 | Physical, mental and cognitive disabilities in relation to utilization of dental care services by nursing home residents | Spec Care Dentist | Cross-sectional study |
| Allen 2022 | The relationship between self-efficacy and oral health status of older adults | Journal of Dentistry | Cross-sectional study |
| Fontanive 2013 | The association between clinical oral health and general quality of life: a population-based study of individuals aged 50-74 in Southern Brazil | Community dentistry and oral epidemiology | Cross-sectional study |
| Forsberg 2008 | Oral health in the adult population of Västerbotten, Sweden--a comparison between an epidemiological survey and data obtained from digital dental records | Swedish dental journal | Epidemiological study |
| De Angelis 2018 | Influence of the oral status on cardiovascular diseases in an older Italian population | International Journal of Immunopathology and Pharmacology | Cross-sectional study |
| Decup 2024 | Risk factors or indicators for dental caries and tooth wear and their relative importance in adults according to age | Journal of Dentistry | Cohort study |
| Decup 2024 | Risk factors or indicators for dental caries and tooth wear and their relative | Journal of Dentistry | Cohort study |
| Freitas 2016 | Oral health status and functional capacity in the elderly: a longitudinal population-based study | Revista brasileira de epidemiologia = Brazilian journal of epidemiology | Cohort study |
| Fries 2024 | A Polygenic Score Predicts Caries Experience in Elderly Swedish Adults | Journal of dental research | Cohort study |
| Fure 2004 | Ten-year cross-sectional and incidence study of coronal and root caries and some related factors in elderly Swedish individuals | Ten-year cross-sectional and incidence study of coronal and root caries and some related factors in elderly Swedish individuals | Cohort study |
| Fure 2003 | Ten-year incidence of tooth loss and dental caries in elderly Swedish individuals | Caries research | Cohort study |
| Furuta 2021 | 10-year trend of tooth loss and associated factors in a Japanese population-based longitudinal study | BMJ Open | Cohort study |
| Gail 2023 | The oral health of adults attending dental practices in England in 2018: a report of a novel method and fndings | British dental journal | Epidemiological study |
| Gaio 2012 | Oral health status in elders from South Brazil: a population-based study | Gerodontology | Cohort study |
| Galesi-Pacheco 2020 | Oral condition of institutionalized older people: influence on anthropometric indicators and food and nutrient intakes | Clinical nutrition ESPEN | Cross-sectional study |
| Gao 2024 | Oral diseases are associated with cognitive function in adults over 60 years old | Oral diseases | Epidemiological study |
| Gao 2020 | The Oral Health Status of Chinese Elderly People with and without Dementia: A Cross-Sectional Study | International journal of environmental research and public health | Cross-sectional study |
| Gao 2018 | How Root Caries Differs between Middle-aged People and the Elderly: Findings from the 4th National Oral Health Survey of China | The Chinese journal of dental research | Cross-sectional study |
| Gao 2018 | Dental Caries in Chinese Elderly People: Findings from the 4th National Oral Health Survey | The Chinese journal of dental research | Epidemiological study |
| Gaskin 2022 | Predictors of Unmet Dental Health Needs in US Adults in 2018: A Cross-Sectional Analysis | JDR clinical and translational research | Cross-sectional study |
| Ghanbari 2024 | Dental caries prevalence in relation to the cardiovascular diseases: cross-sectional findings from the Iranian kurdish population | BMC oral health | Cross-sectional study |
| Ghazal Aarabi 2018 | Oral health and access to dental care - a comparison of elderly migrants and non-migrants in Germany | Ethnicity and Health | Cross-sectional study |
| Ghazimoghadam 2021 | Oral health status in the Pars Cohort Study | Archives of Iranian Medicine | Cross-sectional study |
| Gianopoulos 2014 | Oral health assessment in the San Blas and Santa Ana populations of Nicaragua | International journal of dental hygiene | Cross-sectional study |
| Gil-Montoya 2014 | Atraumatic restorative treatment and Carisolv use for root caries in the elderly: 2-year follow-up randomized clinical trial | Clinical oral investigations | RCT |
| Gilbert 2001 | Twenty-four month incidence of root caries among a diverse group of adults | Caries research | Cohort study |
| D'Alessandro 2018 | Oral Health Status in Alzheimer's Disease Patients: A Descriptive Study in an Italian Population | The Journal of Contemporary Dental Practice | Cross-sectional study |
| Bartoli 2019 | Oral health status as predictor of 1-year mortality after discharge from an acute geriatric unit | European Geriatric Medicine | Cross-sectional study |
| Glowacka 2019 | Needs for gerodontological treatment in the elderly living in Lower Silesia | Dental and Medical Problems | Cross-sectional study |
| Goel 2006 | Oral healthcare for elderly: identifying the needs and feasible strategies for service provision | Indian journal of dental research | Epidemiological study |
| Goergen 2023 | Oral conditions associated with oral health related quality of life: A population-based cross-sectional study in Brazil | Journal of dentistry | Cross-sectional study |
| Gokalp 2010 | National survey of oral health status of children and adults in Turkey | Community dental health | Cross-sectional study |
| Gokalp 2012 | Root caries in 35-44 and 65-74 year-olds in Turkey | Community dental health | Cross-sectional study |
| Carter 2004 | Oral health status and oral treatment needs of dependent elderly people in Christchurch | The New Zealand Medical Journal | Cross-sectional study |
| Gordon 2018 | Use of the Dental Electronic Health Record for Research: Assessing Demographic and Oral Health Characteristics Data for Clinic Patients | Journal of dental education | Cross-sectional study |
| Gostemeyer 2019 | Atraumatic vs conventional restorative treatment for root caries lesions in older patients: Meta- and trial sequential analysis | Gerodontology | Systematic review and/or meta analysis |
| Gousalya 2023 | Effect of fluoride on oral health status among general population residing in high-and low-level fluoride blocks in Erode District, Tamil Nadu, India: A cross-sectional study | Journal of Pharmacy and Bioallied Sciences | Cross-sectional study |
| Gousalya 2023 | An analysis of gender differences and assessment of oral health status among the general population residing in Kanyakumari District: A pathfinding scientific study | Journal of Pharmacy and Bioallied Sciences | Cross-sectional study |
| Griffin 2005 | New coronal caries in older adults: Implications for prevention | Journal of Dental Research | Systematic review and/or meta analysis |
| Griffin 2019 | Changes in Older Adults' Oral Health and Disparities: 1999 to 2004 and 2011 to 2016 | Journal of the American Geriatrics Society | Epidemiological study |
| Guan 2020 | Status of dental caries and associated factors in Tibetan adults: findings from the fourth China National Oral Health Survey | BMC oral health | Cross-sectional study |
| Guerreiro 2024 | Caries Experience before and after COVID-19 Restrictions: An Observational Study | Journal of Clinical Medicine | Cross-sectional study |
| Guerreiro 2023 | Caries Experience and Risk Indicators in a Portuguese Population: A Cross-Sectional Study | International journal of environmental research and public health | Cross-sectional study |
| Aguirre Escobar 2022 | Oral health profile status and treatment needs in the Salvadoran elderly population:a cross-sectional study | BMC Oral Health | Cross-sectional study |
| Gupta 2018 | Disparities in untreated caries among children and adults in the U.S., 2011-2014 | BMC oral health | Cross-sectional study |
| Hadler-Olsen 2021 | Oral health and use of dental services in different stages of adulthood in Norway: a cross sectional study | BMC oral health | Cross-sectional study |
| Hamalainen 2004 | Changes in dental status over 10 years in 80-year-old people: a prospective cohort study | Community dentistry and oral epidemiology | Cohort study |
| Hamasha 2005 | Coronal and root caries in the older Iowans: 9- to 11-year incidence | Special care in dentistry | Cohort study |
| Hamzah 2025 | Dental Caries Risk and Quality of Life of Middle Age and Older Adults during COVID-19 Pandemic in Indonesia | Pesquisa Brasileira em Odontopediatria e Clinica Integrada | Cross-sectional study |
| Handa 2016 | Oral health status of rural and urban population of Gurgaon block, Gurgaon district using WHO assessment form through multistage sampling technique | Journal of Clinical and Diagnostic Research | Cross-sectional study |
| Hariyani 2018 | Root caries incidence and increment in the population - A systematic review, meta-analysis and meta-regression of longitudinal studies | Journal of dentistry | Systematic review and/or meta analysis |
| Hariyani 2018 | Root surface caries among older Australians | Community dentistry and oral epidemiology | Cohort study |
| Hariyani 2019 | The prevalence and severity of root surface caries across Australian generations | Community dentistry and oral epidemiology | Cohort study |
| Hariyani 2017 | Root caries experience among Australian adults | Gerodontology | Epidemiological study |
| Hatipoglu 2011 | The clinical evaluation of the oral status in Alzheimer-type dementia patients | Gerodontology | Cross-sectional study |
| Hayes 2017 | Evaluation of the Cariogram for root caries prediction | Journal of dentistry | Cohort study |
| Hayes 2016 | Evaluation of Biodentine in the Restoration of Root Caries: a Randomized Controlled Trial | JDR clinical and translational research | RCT |
| Hayes 2016 | Risk indicators associated with root caries in independently living older adults | Journal of dentistry | Cohort study |
| He 2024 | Association Between Diabetes Status, Glycaemic Control and Untreated Root Caries Among United States Adults | International dental journal | Cross-sectional study |
| He 2018 | An oral epidemiological comparison of Chinese and New Zealand adults in 2 key age groups | Community dentistry and oral epidemiology | Epidemiological study |
| Heegaard 2011 | The Copenhagen Oral Health Senior Cohort: design, population and dental health | Gerodontology | Cohort study |
| Adam 2006 | The oral health of individuals with dementia in nursing homes | Gerodontology | Cross-sectional study |
| Henriksen 2004 | Dental caries among the elderly in Norway | Acta odontologica Scandinavica | Cross-sectional study |
| Hernández-Palacios 2015 | Relationship between gender, income and education and self-perceived oral health among elderly Mexicans. An exploratory study | Ciencia & saude coletiva | Cross-sectional study |
| Heydari 2024 | The association between dental caries, periodontal diseases, and tooth loss with diabetes mellitus among the elderly population | Journal of diabetes and metabolic disorders | Cross-sectional study |
| Hoeksema 2017 | Oral health status and need for oral care of care-dependent indwelling elderly: from admission to death | Clinical oral investigations | Cohort study |
| Holmen 2012 | Oral status in home-dwelling elderly dependent on moderate or substantial supportive care for daily living: prevalence of edentulous subjects, caries and periodontal disease | Gerodontology | Cross-sectional study |
| Holtzman 2015 | Detection and proportion of very early dental caries in independent living older adults | Lasers in surgery and medicine | Cross-sectional study |
| Hopcraft 2012 | Edentulism and dental caries in Victorian nursing homes | Gerodontology | Epidemiological study |
| Horncastle 2024 | Comparing approaches to determining poor oral health among older adults in a national survey | Gerodontology | Cross-sectional study |
| Hosseinpour Sarmadi 2025 | Dental caries and related factors in the elderly of the Azar cohort population: A cross-sectional study | PloS one | Cross-sectional study |
| Huang 2014 | Late-life depressive disorder, oral health and dental care utilization among U.S. Older adults | Journal of the American Geriatrics Society | Cohort study |
| Huang 2025 | Development of a Nomogram for Root Caries Risk Assessment in a Chinese Elderly Population | Journal of dentistry | Cross-sectional study |
| Hugo 2012 | Depressive symptoms and untreated dental caries in older independently living South Brazilians | Caries research | Cross-sectional study |
| Hugoson 2012 | Dental caries in relation to smoking and the use of Swedish snus: epidemiological studies covering 20 years (1983-2003) | Acta odontologica Scandinavica | Epidemiological study |
| Hugoson 2000 | Caries prevalence and distribution in 20-80-year-olds in Jönköping, Sweden, in 1973, 1983, and 1993 | Community dentistry and oral epidemiology | Cross-sectional study |
| Hugoson 2008 | Thirty year trends in the prevalence and distribution of dental caries in Swedish adults (1973-2003) | Swedish dental journal | Epidemiological study |
| Hung 2024 | The Association of Vitamin D Levels and Dental Caries in Older Adults: A Cross-Sectional Study | Nutrients | Cross-sectional study |
| Hwang 2012 | Relationship between oral impacts on daily performance and chewing ability among independent elders residing in Daejeon City, Korea | Gerodontology | Cross-sectional study |
| Ichikawa 2024 | Low Salivary IgA Levels Against PAc (361-386) as a Risk Factor for Root Caries in Older Adults | Clinical and experimental dental research | Cohort study |
| Ide 2018 | Correlation between dental conditions and comorbidities in an elderly Japanese population: A cross-sectional study | Medicine | Cross-sectional study |
| Iglesias Corchero 2008 | Oral health in people over 64 years of age, institutionalized in Centres for the Aged in the Vigo Health District Spain, 2005 | Medicina oral, patologia oral y cirugia bucal | Cross-sectional study |
| Imazato 2006 | Prevalence of root caries in a selected population of older adults in Japan | Journal of oral rehabilitation | Epidemiological study |
| Islas-Granillo 2012 | Socioeconomic, sociodemographic, and clinical variables associated with root caries in a group of persons age 60years and older in Mexico | Geriatrics and Gerontology International | Cross-sectional study |
| Islas-Granillo 2019 | Indicators of oral health in older adults with and without the presence of multimorbidity: a cross-sectional study | Clinical interventions in aging | Cross-sectional study |
| Jäger 2009 | Oral hygiene in nursing home residents. Impact of an oral health education programme for the nursing personnel on the residents' oral health | Bundesgesundheitsblatt, Gesundheitsforschung, Gesundheitsschutz | Prospective, longitudinal intervention study |
| Jaafar 2014 | Is the burden of oral diseases higher in urban disadvantaged community compared to the national prevalence? | BMC public health | Cross-sectional study |
| Jabir 2022 | Evaluating the effectiveness of fluoride varnish in preventing caries amongst Long-Term Care Facility Residents | Gerodontology | non-RCT |
| Jadhav 2024 | Oral Manifestations in Geriatric Patients: An Observational Study in Western Maharashtra | Cureus | Cross-sectional study |
| Jamalinasab 2024 | Oral Health and Oral Health-Related Quality of Life among Elderly People in Iran | Iranian journal of nursing and midwifery research | Cross-sectional study |
| Janakiram 2018 | Prevalence of dental caries in India among the WHO index age groups: A meta-analysis | Journal of Clinical and Diagnostic Research | Systematic review and/or meta analysis |
| Chalmers 2002 | Caries experience in existing and new nursing home residents in Adelaide, Australia | Gerodontology | Cohort study |
| Chalmers 2003 | Oral diseases and conditions in community-living older adults with and without dementia. | Spec Care Dentist | Cross-sectional study |
| Janssens 2018 | The impact of a preventive and curative oral healthcare program on the prevalence and incidence of oral health problems in nursing home residents | PloS one | Cohort study |
| Janssens 2025 | Changing Oral Health Trajectories: A 10-Year Cross-Sectional Comparison of 2 Domiciliary Dental Care Patient Groups in Nursing Homes | Journal of the American Medical Directors Association | Epidemiological study |
| Janssens 2023 | Clinical and Subjective Oral Health Status of Care Home Residents in Europe: A Systematic Review | Journal of the American Medical Directors Association | Systematic review and/or meta analysis |
| Bharateesh 2014 | Association of Root Caries with Oral Habits in Older Individuals Attending a Rural Health Centre of a Dental Hospital in India | Journal of Clinical and Diagnostic Research | Cross-sectional study |
| Jg 2010 | Predictor model of root caries in older adults: reporting of evidence to the translational evidence mechanism | The open dentistry journal | Statistical modeling study |
| Jiang 2024 | Analysis of factors associated with tooth loss in older adults from 1995 to 2015: a population-based cross-sectional survey in Guangdong, China | Clinical oral investigations | Cross-sectional study |
| Jiang 2022 | Unhealthy oral status contributes to the older patients with cognitive frailty: an analysis based on a 5-year database | BMC geriatrics | Cross-sectional study |
| Joaquim 2010 | A comparison of the dental health of Brazilian and Canadian independently living elderly | Gerodontology | Cross-sectional study |
| Johanson 2009 | Prevalence and incidence of dental caries and related risk factors in 70- to 76-year-olds | Acta odontologica Scandinavica | Cohort study |
| Jones 2004 | Comparison of patient-based oral health outcome measures | Quality of life research | Cross-sectional study |
| Jordan 2019 | Trends in caries experience in the permanent dentition in Germany 1997-2014, and projection to 2030: Morbidity shifts in an aging society | Scientific reports | Cross-sectional study |
| Jurasic 2014 | Fluoride effectiveness in high caries risk and medically complex Veterans | Community dentistry and oral epidemiology | Cohort study |
| Kailembo 2018 | Income and wealth as correlates of socioeconomic disparity in dentist visits among adults aged 20 years and over in the United States, 2011-2014 | BMC oral health | Epidemiological study |
| Kalra 2024 | A Cross-Sectional Study to Assess Diabetes as a Risk Factor for Root Caries Amongst the Elderly Population in Udupi District, Karnataka State, India | Scientific World Journal | Cross-sectional study |
| Kamberi 2016 | Prevalence of Dental Caries in Kosovar Adult Population | International journal of dentistry | Epidemiological study |
| Kang 2022 | Association Between Oral Health and Frailty in Older Korean Population: A Cross-Sectional Study | Clinical interventions in aging | Cross-sectional study |
| Karki 2015 | Oral health status of older people living in care homes in Wales | British dental journal | Cross-sectional study |
| Dahl 2011 | Oral health-related quality of life among adults 68-77 years old in Nord-Trondelag, Norway | International journal of dental hygiene | Cross-sectional study |
| Khoury 2022 | The association between oral health and nutritional status in older adults: a cross-sectional study | BMC geriatrics | Cross-sectional study |
| Kida 2006 | Clinical and socio-behavioral correlates of tooth loss: a study of older adults in Tanzania | BMC oral health | Cross-sectional study |
| Kim 2021 | A decision tree to identify the combinations of non-communicable diseases that constitute the highest risk for dental caries experience: A hospital records-based study | PloS one | Cross-sectional study |
| Kim 2018 | Differences in the oral health status and oral hygiene practices according to the extent of post-stroke sequelae | Journal of oral rehabilitation | Epidemiological study |
| Kim 2017 | Inequality in unmet dental care needs among South Korean adults | BMC oral health | Cross-sectional study |
| Kim 2017 | Body mass index and oral health status in Korean adults: the Fifth Korea National Health and Nutrition Examination Survey | International journal of dental hygiene | Cross-sectional study |
| Avlund 2004 | Tooth loss and caries prevalence in very old Swedish people:the relationship to cognitive function and functional ability | Gerodontology | Cross-sectional study |
| Avlund 2005 | The Strength of Two Indicators of Social Position on Oral Health Among Persons Over the Age of 80 Years | Journal of Public Health Dentistry | Cohort study |
| Kishi 2015 | Oral health-related quality of life and related factors among residents in a disaster area of the Great East Japan Earthquake and giant tsunami | Health and Quality of Life Outcomes | Cross-sectional study |
| Klotz 2020 | Differences Between the Oral Health of People Aged 50 and 70 Years - An Exploratory Cohort Study | Oral health & preventive dentistry | Epidemiological study |
| Kocher 2021 | Trends in dental and oral health status in Germany between 1997 and 2014 | Bundesgesundheitsblatt, Gesundheitsforschung, Gesundheitsschutz | Epidemiological study |
| Konopka 2017 | Oral health parameters in the regional study among young seniors in an urban area of Wroclaw | Dental and Medical Problems | Cross-sectional study |
| Kovac-Kavcic 2001 | The change of DMFT counts in Slovenia | Caries research | Cohort study |
| Krustrup 2007 | Dental caries prevalence among adults in Denmark--the impact of socio-demographic factors and use of oral health services | Community dental health | Cross-sectional study |
| Krustrup 2008 | The overtime effect of social position on dental caries experience in a group of old-aged Danes born in 1914 | Journal of public health dentistry | Cohort study |
| Kularatne 2007 | Root surface caries in older individuals from Sri Lanka | Caries research | Cross-sectional study |
| Kumar 2023 | Assessment of oral health status amongst the Sevayats of Shree Jagannath Temple, Puri, Odisha: A cross sectional study | National journal of maxillofacial surgery | Cross-sectional study |
| Kumara-Raja 2016 | Prevalence of root caries among elders living in residential homes of Bengaluru city, India | Journal of clinical and experimental dentistry | Cross-sectional study |
| Crocombe 2009 | Comparison of adult oral health in Australia, the USA, Germany and the UK | Australian Dental Journal | Cross-sectional study |
| Lam 2009 | Assessment of the use of xerogenic medications for chronic medical and dental conditions among adult day health participants | The Consultant pharmacist | Cross-sectional study |
| Lauritano 2019 | Oral Health Status and Need for Oral Care in an Aging Population: A Systematic Review | International journal of environmental research and public health | Systematic review and/or meta analysis |
| León 2018 | Root caries arrest with high-fluoride dentifrices in community-dwelling elders, RCT | Journal of dentistry | RCT |
| León 2018 | High-fluoride dentifrices in community-dwelling elders, RCT | Journal of dental research | RCT |
| Lee 2025 | Food insecurity and its relationship to mental health and oral health in older adults | Gerodontology | Cross-sectional study |
| Lee 2022 | Relationship between Bone Mineral Density and Dental Caries in Koreans by Sex and Menopausal State | International Journal of Environmental Research and Public Health | Cross-sectional study |
| Leon 2019 | High fluoride dentifrice for preventing and arresting root caries in community-dwelling older adults: A randomized controlled clinical trial | Journal of dentistry | RCT |
| Leon 2016 | Validation of the Spanish version of the oral health impact profile to assess an association between quality of life and oral health of elderly Chileans | Gerodontology | Cross-sectional study |
| Leon, 2019 | Assessment of oral health-related quality of life as a function of non-invasive treatment with high-fluoride toothpastes for root caries lesions in community-dwelling elderly | International dental journal | RCT |
| Crocombe 2013 | Is clinical oral health poorer in regional areas compared with major city areas? | The Australian journal of rural health | Cross-sectional study |
| Liang 2013 | Racial disparities in trajectories of dental caries experience | Community dentistry and oral epidemiology | Cohort study |
| Liang 2014 | Social stratification, oral hygiene, and trajectories of dental caries among old Americans | Journal of aging and health | Cohort study |
| Alvarez 2015 | Dental caries in Uruguayan adults and elders:findings from the first Uruguayan National Oral Health Survey | Cadernos de saude publica | Cross-sectional study |
| Lin 2001 | Coronal and root caries in Southern Chinese adults | Journal of Dental Research | Epidemiological study |
| Linabarger 2021 | Utility of State-Based Basic Screening Survey Reports for National Oral Health Surveillance in Older Adults | Preventing Chronic Disease | Systematic review and/or meta analysis |
| Christensen 2015 | Root caries, root surface restorations and lifestyle factors in adult Danes | Acta Odontologica Scandinavica | Cross-sectional study |
| Liu 2020 | Dental Caries Prediction Based on a Survey of the Oral Health Epidemiology among the Geriatric Residents of Liaoning, China | BioMed Research International | Cross-sectional study |
| Liu 2013 | Prevalence and Correlates of Dental Caries in an Elderly Population in Northeast China | PLoS ONE | Cross-sectional study |
| Liu 2014 | Social disparities in dentition status among American adults | International Dental Journal | Epidemiological study |
| Lo 2006 | ART and conventional root restorations in elders after 12 months | Journal of dental research | RCT |
| Lo 2004 | Oral health status of institutionalised elderly in Hong Kong | Community dental health | Cross-sectional study |
| Lopez 2017 | Ageing, dental caries and periodontal diseases | Journal of Clinical Periodontology | Systematic review and/or meta analysis |
| Lopez 2013 | Comparison between amine fluoride and chlorhexidine with institutionalized elders: a pilot study | Gerodontology | Cross-sectional study |
| de Albuquerque 2023 | Dental Caries, Tooth Loss and Quality of Life of Individuals Exposed to Social Risk Factors in Northeast Brazil | International journal of environmental research and public health | Cross-sectional study |
| Luan 2000 | Ten-Year Incidence of Dental Caries in Adult and Elderly Chinese | Caries research | Cohort study |
| De Visschere 2016 | An oral health survey of vulnerable older people in Belgium | Clinical oral investigations | Cross-sectional study |
| Anderson 2016 | Oral Health Status of Older Adults Attending Senior Centers and Congregate Meal Sites in New Hampshire | Journal of Dental Hygiene | Cross-sectional study |
| Lundegren 2012 | Oral health in the adult population of Skåne, Sweden: a clinical study | Acta odontologica Scandinavica | Cross-sectional study |
| Luo 2007 | ART and conventional root restorations in elders after 3 years | Journal of dental research | RCT |
| Lussi 2001 | Prevalence and risk factors of root caries. A study of residents over 70 years old in the homes for the aged | Schweizer Monatsschrift fur Zahnmedizin = Revue mensuelle suisse d'odonto-stomatologie = Rivista mensile svizzera di odontologia e stomatologia / SSO | Cross-sectional study |
| Roma 2021 | Critical Assessment on Unmet Oral Health Needs and Oral Health-related Quality of Life Among Old Age Home Inhabitants in Karnataka, India | Clinical, cosmetic and investigational dentistry | Cross-sectional study |
| Machado 2017 | Does the Sense of Coherence modifies the relationship of oral clinical conditions and Oral Health-Related Quality of Life? | Quality of life research : an international journal of quality of life aspects of treatment, care and rehabilitation | Cross-sectional study |
| Machado 2020 | Impact of Seniors Centers on oral health-related quality of life of older adults | Revista de saude publica | Cross-sectional study |
| Mack 2004 | Caries and periodontal disease of the elderly in Pomerania, Germany: results of the Study of Health in Pomerania | Gerodontology | Cross-sectional study |
| Madera 2024 | Colombia's Armed Conflict and Dental Caries among Adults | Caries research | Cohort study |
| Madjova 2018 | Oral and dental status of bulgarian patients- a 5-year study | Journal of IMAB - Annual Proceeding (Scientific Papers) | Cross-sectional study |
| Madlena 2008 | Caries prevalence and tooth loss in Hungarian adult population: results of a national survey | BMC public health | Epidemiological study |
| Maia 2021 | Survival of atraumatic restorative treatment restorations in the elderly patients: a systematic review | Brazilian Oral Research | Systematic review and/or meta analysis |
| Maklennan 2024 | Meta-Analysis of Global Distribution of Root-Caries Prevalence in Middle-Aged and Elderly | Caries Research | Systematic review and/or meta analysis |
| Mamai-Homata 2012 | Risk indicators of coronal and root caries in Greek middle aged adults and senior citizens | BMC Public Health | Epidemiological study |
| Marchesan 2020 | Flossing Is Associated with Improved Oral Health in Older Adults | Journal of dental research | Cohort study |
| Cicciu 2012 | Periodontal health and caries prevalence evaluation in patients affected by Parkinson's disease | Parkinson‚Äôs Disease | Cross-sectional study |
| Cicciu 2013 | Relationship between oral health and its impact on the quality life of Alzheimer's disease patients: A supportive care trial | International Journal of Clinical and Experimental Medicine | Cross-sectional study |
| Cornejo 2013 | Oral Health-Related Quality of Life in institutionalized elderly in Barcelona (Spain) | Med Oral Patol Oral Cir Bucal | Cross-sectional study |
| Mariño 2011 | Oral health status and inequalities among ambulant older adults living in central Chile | Community dental health | Cross-sectional study |
| Marino 2007 | Profile of the oral health among ambulant older Greek and Italian migrants living in Melbourne | Australian dental journal | Cross-sectional study |
| Marino 2014 | Factors related to unmet oral health needs in older adults living in Chile | Archives of gerontology and geriatrics | Cross-sectional study |
| Marino 2012 | Oral health in a convenience sample of Chinese older adults living in Melbourne, Australia | International journal of public health | Cross-sectional study |
| Marino 2015 | Prevalence of root caries among ambulant older adults living in central Chile | Gerodontology | Cross-sectional study |
| Marinova-Takorova 2014 | Prevalence of root surface caries in patients from the faculty of dental medicine, sofia, Bulgaria | Archives of the Balkan Medical Union | Epidemiological study |
| Marques 2013 | Root caries prevalence and severity in Brazilian adults and older people | Prevalencia e extensao da carie dentaria radicular em adultos e idosos brasileiros. | Cross-sectional study |
| Martin 2017 | Oral Health Status of Independent Older Adults in Texas: An observational study comparing urban and rural areas | Journal of dental hygiene | Cross-sectional study |
| Martins 2008 | Factors associated to self perceived need of dental care among Brazilian elderly | Revista de saude publica | Cross-sectional study |
| Marwa 2019 | The potential impact of age, gender, body mass index, socioeconomic status and dietary habits on the prevalence of dental caries among Egyptian adults: a cross-sectional study | F1000Research | Cross-sectional study |
| Masood 2017 | The relationship between oral health and oral health related quality of life among elderly people in United Kingdom | Journal of Dentistry | Epidemiological study |
| Matthews 2012 | Oral health status of long-term care residents-a vulnerable population | Journal (Canadian Dental Association) | Cross-sectional study |
| Mattila 2010 | Prevalence and simultaneous occurrence of periodontitis and dental caries | Journal of clinical periodontology | Epidemiological study |
| Maupome 2006 | The relationship between cardiovascular xerogenic medication intake and the incidence of crown/root restorations | Journal of public health dentistry | Cohort study |
| Maupome 2008 | Oral disorders in institution-dwelling elderly adults: a graphic representation | Special care in dentistry : official publication of the American Association of Hospital Dentists, the Academy of Dentistry for the Handicapped, and the American Society for Geriatric Dentistry | Cross-sectional study |
| Mazurkiewicz 2023 | Dietary Habits and Oral Hygiene as Determinants of the Incidence and Intensity of Dental Caries-A Pilot Study | Nutrients | Cross-sectional study |
| McNally 2014 | The oral health of ageing baby boomers: a comparison of adults aged 45-64 and those 65 years and older | Gerodontology | Cross-sectional study |
| Mei Na 2014 | Oral health status and complete denture status of independent-living Singaporean elderly residing in a community home | Singapore dental journal | Cross-sectional study |
| Melo 2016 | Factors associated with negative self-perception of oral health among institutionalized elderly | Ciencia & saude coletiva | Cross-sectional study |
| Mendes 2012 | Analysis of socio-demographic and systemic health factors and the normative conditions of oral health care in a population of the Brazilian elderly | Gerodontology | Cross-sectional study |
| Yotat 2015 | Oral Health Status of the Elderly at Tonga, West Region, Cameroon | International journal of dentistry | Cross-sectional study |
| Milledge 2024 | Cross-Sectional Associations between Nutrient Intake and Tooth Decay in Older Australian Men: The Concord Health and Ageing in Men Project | Caries research | Cross-sectional study |
| Milstein 2000 | Oral health status in an institutionalised elderly Jewish population | journal of the South African Dental Association | Cross-sectional study |
| de Oliveira Miranda 2018 | Caries prevalence among Brazilian indigenous population of urban areas based on the 2010 National Oral Health Survey | Ciencia & saude coletiva | Cross-sectional study |
| Mirza 2024 | Social Disadvantage and Multimorbidity Including Oral Conditions in the United States | Journal of dental research | Cross-sectional study |
| Mitov 2014 | Dental care and treatment needs of elderly in nursing homes in the Saarland: Perceptions and oral health status of the inhabitants | Journal of Public Health | Cross-sectional study |
| Mittal 2022 | Willingness to Pay for Preventive Dental Care Amongst Older Adults | International dental journal | Cross-sectional study |
| Mittal 2021 | Correlation between microbial host factors and caries among older adults | BMC oral health | Cross-sectional study |
| Mohammad 2017 | The Impact of Hyposalivation on Quality of Life (QoL) and Oral Health in the Aging Population of Al Madinah Al Munawarrah | International Journal of Environmental Research and Public Health | Cross-sectional study |
| Molete 2014 | Oral health needs and barriers to accessing care among the elderly in Johannesburg | SADJ : journal of the South African Dental Association | Cross-sectional study |
| Molinari 2007 | Restorative caries therapy in nursing home residents using composite resins and compomers without a rubber dam | Quintessence international | Cohort study |
| Abdel Fattah 2022 | Epidemiology of dental caries in permanent dentition: evidence from a population-based survey in Egypt | BMC Public Health | Epidemiological study |
| Monaghan 2017 | Measuring oral health impact among care home residents in Wales | Community dental health | Cross-sectional study |
| Montal 2006 | Oral hygiene and the need for treatment of the dependent institutionalised elderly | Gerodontology | Cross-sectional study |
| Morén 2023 | The Effect of Domiciliary Professional Oral Care on Root Caries Progression in Care-Dependent Older Adults: A Systematic Review | Journal of clinical medicine | Systematic review and/or meta analysis |
| Morales-Suarez-Varela 2011 | Oral and dental health of non-institutionalized elderly people in Spain | Archives of gerontology and geriatrics | Cross-sectional study |
| Moreira Rda 2005 | Oral health of Brazilian elderly: a systematic review of epidemiologic status and dental care access | Cadernos de saude publica | Systematic review and/or meta analysis |
| Morita 2007 | Is there a gradient by job classification in dental status in Japanese men? | European journal of oral sciences | Cross-sectional study |
| Morse 2002 | Dental caries in persons over the age of 80 living in Kungsholmen, Sweden: findings from the KEOHS project | Community dental health | Cross-sectional study |
| Morse 2002 | Prosthetic crowns and other clinical risk indicators of caries among old-old Swedish adults: findings from the KEOHS Project. Kungsholmen Elders Oral Health Study | Gerodontology | Cross-sectional study |
| Moss 2022 | High sugar intake from sugar-sweetened beverages is associated with prevalence of untreated decay in US adults: NHANES 2013-2016 | Community dentistry and oral epidemiology | Cross-sectional study |
| Msyamboza 2016 | Magnitude of dental caries, missing and filled teeth in Malawi: National Oral Health Survey | BMC oral health | Epidemiological study |
| Amanat 2020 | Dentition status among an ethnically diverse older urban Malaysian population | Journal of Public Health | Cross-sectional study |
| Mulic 2020 | The frequency of enamel and dentin caries lesions among elderly Norwegians | Acta odontologica Scandinavica | Cross-sectional study |
| Muller 2011 | Caries and periodontal disease in patients with Parkinson's disease | Special care in dentistry : official publication of the American Association of Hospital Dentists, the Academy of Dentistry for the Handicapped, and the American Society for Geriatric Dentistry | Cross-sectional study |
| Mungia 2008 | Interaction of age and specific saliva component output on caries | Aging clinical and experimental research | Cross-sectional study |
| Al-Haboubi 2014 | Oral health patterns of independently living dentate older people: a cross-sectional survey of dental attendees in south-east London | Gerodontology | Cross-sectional study |
| Diep 2023 | Caries experience and risk indicators of having decayed teeth among 65-year-olds in Oslo, Norway: a cross-sectional study | BMC Oral Health | Cross-sectional study |
| Buranarom 2020 | Hyposalivation, oral health, and Candida colonization in independent dentate elders | PLOS ONE | Cross-sectional study |
| Nalcaci 2007 | Evaluation of the oral health status of the people aged 65 years and over living in near rural district of Middle Anatolia, Turkey | Archives of gerontology and geriatrics | Cross-sectional study |
| Namal 2008 | Dental health status and risk factors for dental caries in adults in Istanbul, Turkey | Eastern Mediterranean health journal = La revue de sante de la Mediterranee orientale = al-Majallah al-sihhiyah li-sharq al-mutawassit | Cross-sectional study |
| Bashir 2022 | Update on the prevalence of untreated caries in the US adult population, 2017-2020 | Journal of the American Dental Association | Cross-sectional study |
| Nazar 2023 | Association between oral health and cognitive decline in older Chileans | Gaceta sanitaria | Cross-sectional study |
| Nazar 2021 | Oral Health Status among Adult Employees in Kuwait | Oral health & preventive dentistry | Cross-sectional study |
| Neethu 2023 | Association of social network and social support with oral health among institutionalized elderly: A cross-sectional study in Bangalore city | Clinical Epidemiology and Global Health | Cross-sectional study |
| Nelson 2019 | The psychometric properties of a new oral health illness perception measure for adults aged 62 years and older | PloS one | Cross-sectional study |
| Nevalainen 2004 | A 5-year follow-up study on the prosthetic rehabilitation of the elderly in Helsinki, Finland | Journal of Oral Rehabilitation | Cohort study |
| Ngoc 2021 | An Experimental and Clinically Controlled Study of the Prevention of Dental Caries Using 1.23% Fluoride Gel in Elderly Patients | Journal of International Society of Preventive & Community Dentistry | Cohort study |
| Ngoenwiwatkul 2014 | Dental status and its impact on the quality of life of elderly in Phon Sawan, Nakhon Phanom Province | The Southeast Asian journal of tropical medicine and public health | Cross-sectional study |
| Nguyen 2010 | Oral health status of adults in Southern Vietnam - a cross-sectional epidemiological study | BMC oral health | Cross-sectional study |
| Nicolau 2000 | Number of teeth and risk of root caries | Gerodontology | Cross-sectional study |
| Niesten 2016 | Oral health-related quality of life and associated factors in a care-dependent and a care-independent older population | Journal of dentistry | Cross-sectional study |
| Nihtila 2010 | Heavy consumption of dental services among Finnish adults | Community dental health | Cross-sectional study |
| Nordblom 2025 | Cost-effectiveness-analysis of oral health remotivation and reinstruction in nursing homes in a cluster-randomized controlled trial | Journal of dentistry | Cohort study |
| Norderyd 2015 | Oral health of individuals aged 3-80 years in Jonkoping, Sweden during 40 years (1973-2013). II. Review of clinical and radiographic findings | Swedish dental journal | Epidemiological study |
| Norderyd 2015 | Oral health of individuals aged 3-80 years in Jonkoping, Sweden, during 40 years (1973-2013). I. Review of findings on oral care habits and knowledge of oral health | Swedish dental journal | Epidemiological study |
| Northridge 2012 | Tooth loss and dental caries in community-dwelling older adults in northern Manhattan | Gerodontology | Cross-sectional study |
| Novaes 2018 | Factors associated with need for dental treatment: a cross-sectional study at Fluminense Federal University, Rio de Janeiro, Brazil, 2013 | Epidemiologia e servicos de saude : revista do Sistema Unico de Saude do Brasil | Cross-sectional study |
| Nunn 2000 | The condition of teeth in the UK in 1998 and implications for the future | British dental journal | Epidemiological study |
| Okeigbemen 2009 | Prevalence and severity of dental caries among pensioners in Benin City, Nigeria | Odonto-stomatologie tropicale = Tropical dental journal | Cross-sectional study |
| Olerud 2016 | Oral health status in older immigrants in a medium-sized Swedish city | Special care in dentistry : official publication of the American Association of Hospital Dentists, the Academy of Dentistry for the Handicapped, and the American Society for Geriatric Dentistry | Cross-sectional study |
| Oliveira 2020 | Factors associated with oral health-related quality of life of institutionalized elders | Brazilian oral research | Cross-sectional study |
| Oliver 2021 | Should atraumatic restorative treatment be the preferred treatment for older patients? | Evidence-based dentistry | Cohort study |
| Orhan 2024 | Dental caries and associated factors among Turkish children and adults: Findings from the 3rd National Oral Health Survey | Community dentistry and oral epidemiology | Cross-sectional study |
| Ortíz-Barrios 2019 | The impact of poor oral health on the oral health-related quality of life (OHRQoL) in older adults: the oral health status through a latent class analysis | BMC oral health | Cross-sectional study |
| Oscarson 2017 | Is caries equally distributed in adults? A population-based cross-sectional study in Norway - the TOHNN-study | Acta odontologica Scandinavica | Cross-sectional study |
| Oshikohji 2011 | Relationship between receiving a workplace oral health examination including oral health instruction and oral health status in the Japanese adult population | Journal of occupational health | Cross-sectional study |
| D'Avila 2017 | Association between Root Caries and Depressive Symptoms among Elders in Carlos Barbosa, RS, Brazil | Brazilian Dental Journal ( | Cross-sectional study |
| Brandt 2024 | Clinically estimated signs of hyposalivation in older adult residents of long-term care | Gerodontology | Cross-sectional study |
| Oziegbe 2023 | Parity, dental caries and implications for maternal depletion syndrome in northern Nigerian Hausa women | PLoS ONE | Cross-sectional study |
| Andersson 2017 | Dental status in nursing home residents with domiciliary dental care in Sweden | Community Dental Health | Cross-sectional study |
| Pérez-Nicolás 2023 | Use and efficacy of mouthwashes in elderly patients: A systematic review of randomized clinical trials | Annals of anatomy = Anatomischer Anzeiger : official organ of the Anatomische Gesellschaft | Systematic review and/or meta analysis |
| Paiva 2021 | Dental caries experience and its impact on quality of life in Latin American and Caribbean countries | Brazilian oral research | Systematic review and/or meta analysis |
| Patel 2022 | Refocusing dental care: A risk-based preventative oral health programme for dentate older people in UK care homes | Gerodontology | Prospective cohort evaluation |
| De Palma 2005 | Oral health of homeless adults in Stockholm, Sweden | Acta Odontologica Scandinavica | Cross-sectional study |
| Patro 2008 | Prevalence of dental caries among adults and elderly in an urban resettlement colony of New Delhi | Indian journal of dental research : official publication of Indian Society for Dental Research | Cross-sectional study |
| Paulander 2003 | Association between level of education and oral health status in 35-, 50-, 65- and 75-year-olds | Journal of clinical periodontology | Epidemiological study |
| Pawinska 2023 | Dental Status and Oral Health Behaviors of Selected 45-74-Year-Old Men from Northeastern Poland | International journal of environmental research and public health | Cross-sectional study |
| Peres 2020 | Trend and distribution of coronal dental caries in Australians adults | Australian dental journal | Cross-sectional study |
| Petelin 2012 | Oral health of the elderly living in residential homes in Slovenia | Gerodontology | Cross-sectional study |
| Petersson 2004 | Comparing caries risk factors and risk profiles between children and elderly | Swedish dental journal | Cohort study |
| Pham 2018 | Dental and periodontal problems of elderly people in Vietnamese nursing homes | Gerodontology | Cross-sectional study |
| Philip 2012 | Caries experience of institutionalized elderly and its association with dementia and functional status | International journal of dental hygiene 2012 Vol. 10 Issue 2 Pages | Cohort study |
| Allenspach 2024 | Oral health status of institutionalized older adults receiving domiciliary dental care: A cross-sectional retrospective study | Spec Care Dentist | Cross-sectional study |
| Baca 2009 | Effect of chlorhexidine-thymol varnish on root caries in a geriatric population: A randomized double-blind clinical trial | Journal of dentistry | Cohort study |
| Pitchika 2021 | Impact of Powered Toothbrush Use and Interdental Cleaning on Oral Health | Journal of dental research | Cross-sectional study |
| Pitchika 2022 | Factors influencing tooth loss in European populations | Journal of clinical periodontology | Cross-sectional study |
| Pizarro 2006 | Dental health differences by social class in home-dwelling seniors of Barcelona, Spain | Journal of public health dentistry | Cross-sectional study |
| Ploysangngam 2008 | Oral health status in the elderly priests in Bangkok | Journal of the Medical Association of Thailand | Cross-sectional study |
| Qin 2021 | Greater inequalities in dental caries treatment than in caries experience: a concentration index decomposition approach | BMC oral health | Cross-sectional study |
| Chang 2023 | Decomposing socioeconomic inequalities in dental caries among Chinese adults: findings from the 4th national oral health | BMC Oral Health | Epidemiological study |
| Quinteros 2014 | Caries experience and use of dental services in rural and urban adults and older adults from central Chile | International dental journal | Cross-sectional study |
| Rabiei 2010 | Prevalence of oral and dental disorders in institutionalised elderly people in Rasht, Iran | Gerodontology | Cross-sectional study |
| Rabiei 2019 | Dental status of the Iranian elderly: A systematic review and meta-analysis | Journal of investigative and clinical dentistry | Systematic review and/or meta analysis |
| Rachmawati 2025 | Caries risk factors among Indonesian adults: insights from the national health survey | Brazilian oral research | Cross-sectional study |
| Rani 2024 | Oral Health Status, Dietary Intake, and Oral Health-related Quality of Life among Institutionalized Elderly in Bangalore | Journal of pharmacy & bioallied sciences | Cross-sectional study |
| Rapp 2021 | Oral Health and Undernutrition in the Frail Elderly Persons | The journal of nutrition, health & aging | Cross-sectional study |
| Ratnayake 2022 | Clinical and Microbiological Evaluation of a Chlorhexidine-Modified Glass Ionomer Cement (GIC-CHX) Restoration Placed Using the Atraumatic Restorative Treatment (ART) Technique | Materials | Cohort study |
| Ravi 2019 | Relationship between geriatric oral health assessment index (GOHAI) and oral health status of the institutionalized elderly in Mangalore, India | Indian Journal of Public Health Research and Development | Cross-sectional study |
| Castrejón-Pérez 2010 | Validation of an instrument for measuring the effects of Oral Health on the quality of life of older adults in Mexico | Pan American journal of public health | Epidemiological study |
| Rejnefelt 2006 | Oral health status in individuals with dementia living in special facilities | International journal of dental hygiene | Systematic review and/or meta analysis |
| Ribeiro Gaião 2009 | Poor dental status and oral hygiene practices in institutionalized older people in northeast Brazil | International journal of dentistry | Cross-sectional study |
| Rihs 2009 | Dental caries in an elderly population in Brazil | Journal of applied oral science : revista FOB | Cross-sectional study |
| Rihs 2008 | Root caries in areas with and without fluoridated water at the Southeast region of São Paulo State, Brazil | Journal of applied oral science : revista FOB | Cross-sectional study |
| Rihs 2005 | Dental root surface caries prevalence among adults and senior citizens in southeast São Paulo State, Brazil | Cadernos de saude publica | Cross-sectional study |
| Ritter 2012 | Risk indicators for the presence and extent of root caries among caries-active adults enrolled in the Xylitol for Adult Caries Trial (X-ACT) | Clinical oral investigations | Cross-sectional study |
| Berg 2000 | Oral health status of older adujts in Arizona:results fromt he Arizona elder study | Speclal Care In Dentistry | Cross-sectional study |
| Borg-Bartolo 2021 | Delivery of mobile dental services to dependent elderly people: results from a pilot study in rural Switzerland | Swiss Dental Journal | Cross-sectional study |
| Roccuzzo 2023 | Evaluation of the Oral Health Conditions and Oral Health-Related Quality of Life in a Community-Dwellers Population Aged >= 45 Years in the Canton of Bern: A Preliminary Pilot Study | International journal of environmental research and public health | Cross-sectional study |
| Rodrigues 2012 | Relation between oral health and nutritional condition in the elderly | Journal of applied oral science : revista FOB | Cross-sectional study |
| Rodseth 2024 | Exploring socioeconomic inequality in caries experience in an adult Norwegian population; the HUNT4 Oral Health Study | Community dentistry and oral epidemiology | Cross-sectional study |
| Rodseth 2023 | Dental caries in a Norwegian adult population, the HUNT4 oral health study; prevalence, distribution and 45-year trends | Acta odontologica Scandinavica | Epidemiological study |
| Agrawal 2015 | Assessment of Dental Caries and Periodontal Disease Status among Elderly Residing in | Journal of International Oral Health | Cross-sectional study |
| Bastos 2010 | Dental caries profile in Monte Negro, Amazonian state of Rondonia, Brazil, in 2008 | Journal of applied oral science | Epidemiological study |
| Rosa 2020 | Important oral care needs of older French people: A cross-sectional study | Revue d'epidemiologie et de sante publique | Cross-sectional study |
| Rothen 2014 | Oral hygiene behaviors and caries experience in Northwest PRECEDENT patients | Community dentistry and oral epidemiology | Cross-sectional study |
| Brailsford 2002 | The effects of the combination of chlorhexidine/thymol- and fluoride-containing varnishes on the severity of root caries lesions in frail institutionalised elderly people | Journal of Dentistry | RCT |
| Brailsford 2002 | The effects of the combination of chlorhexidine/thymol- and | Journal of dentistry | Cohort study |
| Sá 2012 | Oral health conditions of the elderly at the Lar Samaritano long-term-care facility in São Gonçalo-RJ | Ciencia & saude coletiva 2012 | Cross-sectional study |
| Sánchez-García 2007 | Oral health service utilization by elderly beneficiaries of the Mexican Institute of Social Security in México city | BMC health services research | Cross-sectional study |
| Sánchez-García 2014 | Oral health status in older adults with social security in Mexico City: Latent class analysis | Journal of clinical and experimental dentistry | Cross-sectional study |
| Ayub 2024 | Impact of dental caries on the daily lives of geriatric patients visiting dental hospitals in Rawalpindi, Pakistan | Journal of dental research, dental clinics, dental prospects | Cross-sectional study |
| Saarela 2022 | Oral hygiene and health-related quality of life in institutionalized older people | European geriatric medicine | Cross-sectional study |
| Sabbah 2015 | The role of behaviour in inequality in increments of dental caries among Finnish adults | Caries research | Cohort study |
| Safak 2023 | The relationship between oral health and sleep quality in community-dwelling older adults | Nigerian journal of clinical practice | Cross-sectional study |
| Saintrain 2018 | Older adults' dependence in activities of daily living: Implications for oral health | Public health nursing (Boston, Mass.) | Cross-sectional study |
| Salih 2022 | Oral health status and associated factors among Sudanese older adults: A cross-sectional study | Gerodontology | Cross-sectional study |
| Salunke 2019 | Prevalence of dental caries, oral health awareness and treatment-seeking behavior of elderly population in rural Maharashtra | Indian journal of dental research : official publication of Indian Society for Dental Research | Cross-sectional study |
| Samietz 2025 | Dental and oral health assessments in the German National Cohort (NAKO) | BMC oral health 2025 | Epidemiological study |
| Samnieng 2013 | The relationship between seven health practices and oral health status in community-dwelling elderly Thai | Gerodontology | Cross-sectional study |
| Sampaio 2010 | Dental caries and treatment needs among indigenous people of the Potiguara Indian reservation in Brazil | Revista panamericana de salud publica = Pan American journal of public health | Cross-sectional study |
| Samson 2008 | Change in oral health status among the institutionalized Norwegian elderly over a period of 16 years | Acta odontologica Scandinavica | Cohort study |
| Sanchez-Garcia 2010 | Psychometric properties of the General Oral Health Assessment Index (GOHAI) and dental status of an elderly Mexican population | Journal of Public Health Dentistry | Cross-sectional study |
| Sanchez-Garcia 2011 | A prediction model for root caries in an elderly population | Community dentistry and oral epidemiology | Cohort study |
| Andari 2022 | Oral health status in a Lebanese geriatric population | Eastern Mediterranean health journal | Cross-sectional study |
| Santucci 2015 | The Oral Health-Related Quality of Life in State Institutionalized Older Adults in Malta | The International journal of prosthodontics | Cross-sectional study |
| Santucci 2015 | The oral health of institutionalized older adults in Malta | The International journal of prosthodontics | Cross-sectional study |
| Saotome 2006 | Relationship of cariogenic bacteria levels with periodontal status and root surface caries in elderly Japanese | Gerodontology | Cross-sectional study |
| Sarhan 2024 | Fast food consumption and its relationship with oral health among US adults: A cross-sectional NHANES-based study | Saudi Dental Journal | Cross-sectional study |
| Sarita 2004 | Decayed/missing/filled teeth and shortened dental arches in Tanzanian adults | The International journal of prosthodontics | Cross-sectional study |
| Saub 2001 | Dental needs of elderly hostel residents in inner Melbourne | Australian dental journal | Cross-sectional study |
| Saxena 2020 | Assessing the effect of oral diseases on oral health related quality of life of institutionalized elderly using Oral Health Impact Profile (OHIP-14) questionnaire: a pilot study | Roczniki Panstwowego Zakladu Higieny | Cross-sectional study |
| Schiffner 2009 | Oral health in German children, adolescents, adults and senior citizens in 2005 | Community dental health | Epidemiological study |
| Schmalz 2021 | Oral Health-Related Quality of Life, Oral Conditions, and Risk of Malnutrition in Older German People in Need of Care-A Cross-Sectional Study | Journal of clinical medicine | Cross-sectional study |
| Schmoeckel 2021 | Ten-year trends in DMF-S and DMF-T in a northeast German adult population | Journal of dentistry | Cross-sectional study |
| Schmolinsky 2019 | Diabetes status affects long-term changes in coronal caries - The SHIP Study | Scientific reports | Cohort study |
| Schwendicke 2017 | Cost-effectiveness of root caries preventive treatments | Journal of dentistry | cost-effectiveness analysis |
| Schwendicke 2018 | Root caries experience in Germany 1997 to 2014: Analysis of trends and identification of risk factors | Journal of dentistry | Epidemiological study |
| Sekar 2019 | Assessment of oral health status for formulation of draft oral health policy for the residents of chennai city, tamil nadu - a pilot study | Indian Journal of Public Health Research and Development | Cross-sectional study |
| Sekundo 2020 | Oral health and functional capacity of centenarians | Scientific reports | Cross-sectional study |
| Seoane 2023 | Residual dentition, prosthesis type and oral health-related quality of life in Uruguayan adults | Gerodontology | Cross-sectional study |
| Shah 2003 | Gender issues and oral health in elderly Indians | International dental journal | Cross-sectional study |
| Shah 2004 | Impact of socio-demographic variables, oral hygiene practices, oral habits and diet on dental caries experience of Indian elderly: a community-based study | Gerodontology | Cross-sectional study |
| Shao 2018 | Socio-demographic factors, dental status and health-related behaviors associated with geriatric oral health-related quality of life in Southwestern China | Health and quality of life outcomes | Cross-sectional study |
| Sheng 2018 | Correlation between oral health and quality of life among the elderly in Southwest China from 2013 to 2015 | Medicine | Cross-sectional study |
| Shetty 2023 | Prevalence of root caries in Indian population - a systematic review and meta-analysis | Evidence-based dentistry | Systematic review and/or meta analysis |
| Shi 2023 | Association of Interdental Cleaning and Untreated Root Caries in Adults in the United States of America | International dental journal | Systematic review and/or meta analysis |
| Shimazaki 2004 | Relationship between dental care and oral health in institutionalized elderly people in Japan | Journal of oral rehabilitation | Cohort study |
| Shoaee 2024 | National and Subnational Trend of Dental Caries of Permanent Teeth in Iran, 1990-2017 | International dental journal | Systematic review and/or meta analysis |
| Shoaee 2024 | Dental Caries among the Elderly in Iran: A Meta-analysis | Medical journal of the Islamic Republic of Iran | Systematic review and/or meta analysis |
| Silva 2004 | Oral health in adults and the elderly in Rio Claro, São Paulo, Brazil | Cadernos de saude publica | Cross-sectional study |
| Silva 2021 | Impact of oral health on nutritional status, self-perception of oral health and quality of life of institutionalized elderly | Journal of clinical and experimental dentistry | Cross-sectional study |
| Silva 2014 | Dental caries in Victorian nursing homes | Australian dental journal | Cross-sectional study |
| Silva 2008 | Dental caries in a riverine community in Rondônia State, Amazon Region, Brazil, 2005-2006 | Cadernos de saude publica | Cohort study |
| Correa Da Silva 2001 | Self-perception of oral health status by the elderly | Rev Saude Publica | Epidemiological study |
| Simons 2001 | Relationship between oral hygiene practices and oral status in dentate elderly people living in residential homes | Community dentistry and oral epidemiology | Cross-sectional study |
| Simunkovic 2005 | Oral health among institutionalised elderly in Zagreb, Croatia | Gerodontology | Cross-sectional study |
| Singh 2016 | Geriatric oral health predicaments in New Delhi, India | Geriatrics & gerontology international | Cross-sectional study |
| Singh 2024 | Associations between disability type and untreated dental decay among community dwelling US adults | Journal of public health dentistry | Cross-sectional study |
| Sinor 2018 | Main medical illness, oral health complaints and treatment in elderly patients attending Hospital Universiti Sains Malaysia (HUSM) Dental Clinic | International Medical Journal | Cross-sectional study |
| Siukosaari 2005 | Level of education and incidence of caries in the elderly: a 5-year follow-up study | Gerodontology | Cohort study |
| Skaleric 2020 | A Longitudinal Study of DMFT Counts in a Population of Ljubljana Over a Thirty Year Period | Oral health & preventive dentistry | Epidemiological study |
| Skośkiewicz-Malinowska 2021 | Gender-wise comparison of oral health quality of life and its relationship with oral health parameters among elderly from Wroclaw, south-west Poland | PloS one | Cross-sectional study |
| Skośkiewicz-Malinowska 2018 | Oral health condition and occurrence of depression in the elderly | Medicine | Cross-sectional study |
| Skudutyte 2000 | Dental caries in adult Lithuanians | Acta odontologica Scandinavica | Cross-sectional study |
| Slade 2013 | Effects of fluoridated drinking water on dental caries in Australian adults | Journal of Dental Research | Cross-sectional study |
| Critén 2024 | Oral Health Status at Age 60 and 72 Years-A Longitudinal Study | International journal of dental hygiene | Cohort study |
| Smith 2007 | Oral health in rural and remote Western Australian indigenous communities: a two-year retrospective analysis of 999 people | International dental journal | Retrospective cross-sectional analysis |
| Chaisupamongkollarp 2008 | Caries and Periodontal Experience among 998 Priests and Novices in Bangkok | Journal of the Medical Association of Thailand | Cross-sectional study |
| Song-Yu 2012 | Postural stability and occlusal status among Japanese elderly | Gerodontology | Cross-sectional study |
| Arpin 2008 | Dental Caries, Problems Perceived and Use of Services among Institutionalized Elderly in 3 Regions of Quebec, Canada | Journal (Canadian Dental Association) | Cross-sectional study |
| Souza 2018 | Contextual and Individual Determinants of Root Caries in Older People | Caries research | Cross-sectional study |
| Splieth 2004 | Prevalence and distribution of root caries in Pomerania, North-East Germany | Caries research | Cross-sectional study |
| Srisilapanan 2013 | Oral health status of dementia patients in Chiang Mai Neurological Hospital | Journal of the Medical Association of Thailand = Chotmaihet thangphaet | Cross-sectional study |
| Srivastava 2013 | Prevalence of dental caries and periodontal diseases, and their association with socio-demographic risk factors among older persons in Delhi, India: a community-based study | The Southeast Asian journal of tropical medicine and public health | Cross-sectional study |
| Srivastava 2012 | High burden of dental caries in geriatric population of India: a systematic review | Indian journal of public health | Systematic review and/or meta analysis |
| Stangvaltaite-Mouhat 2024 | Co-occurrence of dental caries and periodontitis: multilevel modelling approach | BMC oral health | Cross-sectional study |
| Stangvaltaite-Mouhat 2023 | The 20-Year Trends in Caries and Associated Determinants among Adults in Post-Soviet Lithuania: Repeated Cross-Sectional Studies | Caries research | Cross-sectional study |
| Steele 2007 | ART for treating root caries in older people | Evidence-based dentistry | RCT |
| Steele 2001 | Clinical and behavioural risk indicators for root caries in older people | Gerodontology | Epidemiological study |
| Stephens 2019 | Oral health status of adults in North Carolina assisted living facilities | Journal of public health dentistry | Epidemiological study |
| Stromberg 2012 | Oral status, oral hygiene habits and caries risk factors in home-dwelling elderly dependent on moderate or substantial supportive care for daily living | Community dentistry and oral epidemiology | Epidemiological study |
| Stromberg 2013 | Oral health-related quality-of-life in homebound elderly dependent on moderate and substantial supportive care for daily living | Acta odontologica Scandinavica | Epidemiological study |
| Stubbs 2002 | Dental screening of older adults living in residential aged care facilities in Perth | Australian dental journal | Epidemiological study |
| Sugihara 2010 | Factors associated with root surface caries in elderly | The Bulletin of Tokyo Dental College | Cross-sectional study |
| Sukumar 2025 | Tooth morbidity and its impact on oral related quality of life in elderly tribal population-The Irula experience | BMC oral health | Cross-sectional study |
| Swoboda 2006 | Predictors of oral health quality of life in older adults | Special care in dentistry | Epidemiological study |
| Syrjala 2012 | Dementia and oral health among subjects aged 75 years or older | Gerodontology | Epidemiological study |
| Syrjala 2007 | Relationship between cognitive impairment and oral health: results of the Health 2000 Health Examination Survey in Finland | Acta odontologica Scandinavica | Epidemiological study |
| Taghipour 2016 | National and sub-national drinking water fluoride concentrations and prevalence of fluorosis and of decayed, missed, and filled teeth in Iran from 1990 to 2015: a systematic review | Environmental science and pollution research international | Epidemiological study |
| Taiwo 2007 | Dental caries experience among the elderly population in South East local government area in Ibadan, Nigeria | Odonto-stomatologie tropicale = Tropical dental journal | Epidemiological study |
| Takano 2003 | Factors associated with root caries incidence in an elderly population | Community dental health | Epidemiological study |
| Takata 2009 | Cognitive function and number of teeth in a community-dwelling elderly population without dementia | Journal of oral rehabilitation | Epidemiological study |
| Takiguchi 2016 | Oral health and depression in older Japanese people | Gerodontology | Epidemiological study |
| de Oliveira 2013 | Socio-demographic factors and oral health conditions in the elderly:a population based study | Archives of Gerontology and Geriatrics | Cross-sectional study |
| Tan 2014 | Risk indicators for root caries in institutionalized elders | Community dentistry and oral epidemiology | Cross-sectional study |
| Tan 2010 | A randomized trial on root caries prevention in elders | Journal of dental research | RCT |
| Tanaka 2024 | Oral status of older adults receiving home medical care: A cross-sectional study | Geriatrics & gerontology international | Cross-sectional study |
| Tapias Perdigon 2019 | Oral health assessment of independent elders in Texas | Special care in dentistry | Cross-sectional study |
| Bramantoro 2020 | Relationship between dental caries prevalence and elderly's quality of life | Indian Journal of Public Health Research & Development | Cross-sectional study |
| Tepox-Puga 2023 | Prevalence, and Risk Indicators of Coronal and Root Caries in Mexican Older Adults in Nursing Homes | Clinical, cosmetic and investigational dentistry | Cross-sectional study |
| Thearawiboon 2024 | The role of dentate status and dental caries on diabetes-related complications: a hospital-based cross-sectional study | Journal of medicine and life | Cross-sectional study |
| Thomson 2018 | Oral status, cognitive function and dependency among New Zealand nursing home residents | Gerodontology | Epidemiological study |
| Thomson 2002 | Is medication a risk factor for dental caries among older people? | Community dentistry and oral epidemiology | Cohort study |
| Thorstensson 2009 | Does oral health say anything about survival in later life? Findings in a Swedish cohort of 80+ years at baseline | Community dentistry and oral epidemiology | Cohort study |
| Thorstensson 2003 | Oral health in a population-based sample of the oldest-old: findings in twins 80 years and older in Sweden | Swedish dental journal | Cohort study |
| Thorstensson 2010 | Why do some people lose teeth across their lifespan whereas others retain a functional dentition into very old age? | Gerodontology | Cohort study |
| Thwin 2023 | Dental caries in the Myanmar population: Findings from the first national oral health survey in 2016-2017 | Community dentistry and oral epidemiology | Cross-sectional study |
| Tian 2024 | Cross-sectional survey and analysis of factors influencing the prevalence of dental caries among older individuals aged 65-74 in Guangdong Province in 2021 | BMC Oral Health | Cross-sectional study |
| Tiisanoja 2017 | Sedative load, carious teeth and infection in the periodontium among community-dwelling older people | Gerodontology | Epidemiological study |
| Tokumoto 2022 | Risk factors for root caries annual incidence and progression among older people requiring nursing care: A one-year prospective cohort study | Journal of prosthodontic research | Prospective cohort study |
| Tomson 2015 | West Midlands Care Home Dental Survey 2011: part 2. Results of clinical survey of care home residents | British dental journal | Epidemiological study |
| Tong 2021 | Five-year Survival Rate of Bonded Dental Restorations in Frail Older Adults | JDR clinical and translational research | Retrospective cohort study with longitudinal follow-up |
| Alstad 2008 | Associations between oral sugar clearance, dental caries, and related factors among 71-year-old | Acta Odontologica Scandinavica | Cross-sectional study |
| Tsakos 2004 | The relationship between clinical dental status and oral impacts in an elderly population | Oral health & preventive dentistry | Cross-sectional study |
| Tsakos 2013 | Social relationships and oral health among adults aged 60 years or older | Psychosomatic medicine | Epidemiological study |
| Tsitaishvili 2014 | Assessment of caries prevalence and related risk factors among the adult population of georgia by age and gender | Dental and Medical Problems | Epidemiological study |
| Cicek 2024 | Determining the relationship between oral health and physical function in elderly people | Journal of oral rehabilitation | Epidemiological study |
| Tuuliainen 2020 | Oral Health and Hygiene and Association of Functional Ability: A Cross-Sectional Study Among Old Home Care Clients | Oral health & preventive dentistry | Cross-sectional study |
| Twetman 2004 | Caries-preventive effect of sodium fluoride mouthrinses: a systematic review of controlled clinical trials | Acta odontologica Scandinavica | Systematic review and/or meta analysis |
| Uludamar 2011 | Oral health status and treatment requirements of different residential homes in Istanbul: a comparative study | Archives of gerontology and geriatrics | Epidemiological study |
| Unluer 2007 | Oral health status of the elderly in a residential home in Turkey | Gerodontology | Epidemiological study |
| Urzua 2012 | Dental caries prevalence and tooth loss in chilean adult population: first national dental examination survey | International journal of dentistry | Epidemiological study |
| Chikte 2020 | Dental caries in a South African adult population: findings from the Cape Town Vascular and Metabolic Health Study | International Dental Journal | Cross-sectional study |
| Usuga-Vacca 2021 | Association between root/coronal caries and individual factors in institutionalised elderly using ICDAS severity and activity | BMC oral health | Cross-sectional study |
| Anu 2020 | Oral healthcare needs of the institutionalized ageing population in Tamil Nadu | THE NATIONAL MEDICAL JOURNAL OF INDIA | Cross-sectional study |
| Beltran 2023 | Tooth Loss and Caries Experience of Elderly Chileans in the Context of the COVID-19 Pandemic in Five Regions of Chile | International Journal of Environmental Research and Public Health | Cross-sectional study |
| Valdez 2020 | Frailty and oral health: Findings from the Concord Health and Ageing in Men Project | Gerodontology | Epidemiological study |
| van de Rijt 2020 | The Influence of Oral Health Factors on the Quality of Life in Older People: A Systematic Review | The Gerontologist | Systematic review and/or meta analysis |
| van Kuijk 2021 | Dentition and nutritional status of aged New Zealanders living in aged residential care | Oral diseases | Cross-sectional study |
| Vandenbulcke 2025 | Oral health of nursing home residents in Flanders, Belgium, and its associated factors | Scientific reports | Cross-sectional study |
| Vargas 2003 | Oral health status of older rural adults in the United States | Journal of the American Dental Association | Cross-sectional study |
| Velázquez-Olmedo 2021 | Oral health condition and development of frailty over a 12-month period in community-dwelling older adults | BMC oral health | Cohort study |
| Verma 2019 | Translation and validation of hindi version of oral health impact profile-14, a measure of oral health-related quality of life of geriatrics | Indian journal of dental research | Cross-sectional study |
| Vermaire 2019 | Socioeconomic differences in oral health outcomes among adults in the Netherlands | Nederlands tijdschrift voor tandheelkunde | Cross-sectional study |
| Bansal 2010 | Assessment of oral health status and treatment needs of elders associated with elders' homes of Ambala division, Haryana, India | Indian Journal of Dental Research | Cross-sectional study |
| Vilstrup 2007 | Dental status and dental caries in 85-year-old Danes | Gerodontology | Cross-sectional study |
| Vrbic 2000 | Reasons for the caries decline in Slovenia | Community dentistry and oral epidemiology | Epidemiological study |
| Vu 2023 | Diabetes and dental caries in US adults: an analysis of data from the National Health and Nutrition Examination Survey, 2011-2018 | Community dental health | Cross-sectional study |
| Vucićević-Boras 2002 | Dental health of elderly in retirement homes of two cities in south Croatia--a cross-sectional study | European journal of medical research | Cross-sectional study |
| Wang 2017 | Association between socio-economic status and dental caries in elderly people in Sichuan Province, China: a cross-sectional study | BMJ open | Cross-sectional study |
| Warren 2000 | Dental caries prevalence and dental care utilization among the very old | Journal of the American Dental Association | Cross-sectional study |
| Chen 2023 | The prevalence and common risk indicators of root caries and oral health service utilization pattern among adults, a cross-sectional study | Peer J | Cross-sectional study |
| Wen 2025 | Factors associated with the success of restorative treatment for root caries: A systematic review with meta-analysis | Journal of dentistry | Systematic review and/or meta analysis |
| Wiener 2012 | Hygiene self-care of older adults in West Virginia: effects of gender | Journal of dental hygiene | Epidemiological study |
| Wong 2019 | Oral Health and Its Associated Factors Among Older Institutionalized Residents-A Systematic Review | International journal of environmental research and public health | Systematic review and/or meta analysis |
| Wright 2020 | Associations between oral health and depressive symptoms: Findings from the Concord Health and Ageing in Men Project | Australasian journal on ageing | Epidemiological study |
| Wright 2018 | Oral health of community-dwelling older Australian men: the Concord Health and Ageing in Men Project (CHAMP) | Australian dental journal | Epidemiological study |
| Wu 2011 | Oral health among white, black, and Mexican-American elders: an examination of edentulism and dental caries | Journal of public health dentistry | Epidemiological study |
| Wu 2023 | Influencing factors associated with oral health among older hospitalized patients with ischemic stroke: A cross-sectional survey | International journal of nursing sciences | Cross-sectional study |
| Wu 2025 | Association of oral health status with overweight and obesity in older adults aged ≥ 65 years- evidence from Chinese communities | BMC public health | Cohort study |
| Wu 2018 | Oral Health Indicators for Risk of Malnutrition in Elders | The journal of nutrition, health & aging | Cross-sectional study |
| Wyatt 2002 | Elderly Canadians residing in long-term care hospitals: Part II. Dental caries status | Journal (Canadian Dental Association) | Cross-sectional study |
| Wyatt 2007 | Chlorhexidine and preservation of sound tooth structure in older adults: A placebo-controlled trial | Caries Research | Cohort study |
| Wyatt 2004 | Caries management for institutionalized elders using fluoride and chlorhexidine mouthrinses | Community dentistry and oral epidemiology | Cohort study |
| Chen 2013 | Dental Caries in Older Adults in the Last Year of Life | Journal of the American Geriatrics Society | Cross-sectional study |
| Chen 2011 | Multidimensional Risk Assessment for Tooth Loss in a Geriatric Population with Diverse Medical and Dental Backgrounds | Journal of the American Geriatrics Society | Cohort study |
| Chen 2013 | Oral health in older adults with dementia living in different environments: a propensity analysis | Special Care Dentistry | Cross-sectional study |
| Chen 2015 | Cognitive Impairment, Oral Self-care Function and Dental Caries Severity in Community-dwelling Older Adults | Gerodontolog | Cross-sectional study |
| Yang 2023 | Outreach dental care program for older adults using residential care or day care services in Hong Kong: A cohort study | Community dentistry and oral epidemiology | Cohort study |
| Yang 2015 | Comparison of immunological and microbiological characteristics in children and the elderly with or without dental caries | European journal of oral sciences | Cohort study |
| Ye 2024 | The association between oral health and mild cognitive impairment in community-dwelling older adults | Frontiers in public health | Cross-sectional study |
| Yin 2017 | Oral health status in Sichuan Province: findings from the oral health survey of Sichuan, 2015-2016 | International journal of oral science | Epidemiological study |
| Yoshihara 2020 | The relationship between sucrose intake in coffee or tea, and root or coronal caries in an elderly Japanese population | Community dental health | Cohort study |
| Yoshihara 2009 | A longitudinal study of the relationship between diet intake and dental caries and periodontal disease in elderly Japanese subjects | Gerodontology | Cohort study |
| Yu 2008 | Impact of oral health status on oral health-related quality of life in Chinese hospitalised geriatric patients | Quality of life research | Epidemiological study |
| Yu 2023 | Oral Health and Mortality Among Older Adults: A Doubly Robust Survival Analysis | American journal of preventive medicine | Epidemiological study |
| Yu 2021 | The relationship between different types of caries and periodontal disease severity in middle-aged and elderly people: findings from the 4th National Oral Health Survey of China | BMC oral health | Epidemiological study |
| Zanella-Calzada 2018 | A Case⁻Control Study of Socio-Economic and Nutritional Characteristics as Determinants of Dental Caries in Different Age Groups, Considered as Public Health Problem: Data from NHANES 2013⁻2014 | International journal of environmental research and public health | Epidemiological study |
| Zhang 2020 | Risk indicators for root caries in older adults using long-term social care facilities in Hong Kong | Community dentistry and oral epidemiology | Epidemiological study |
| Zhang 2019 | Risk predictors of dental root caries: A systematic review | Journal of dentistry | Systematic review and/or meta analysis |
| Zhang 2016 | Dental status of an institutionalized elderly population of 60 years and over in Qingdao, China | Clinical oral investigations | Epidemiological study |
| Zhang 2011 | Dental and prosthodontic status of an over 40 year-old population in Shandong Province, China | BMC public health | Epidemiological study |
| Zhang 2013 | Silver diamine fluoride and education to prevent and arrest root caries among community-dwelling elders | Caries Research | RCT |
| Zhao 2011 | Clinical and socio-demographic factors influencing the oral health-related quality of life of Chinese elders | Community dental health | Epidemiological study |
| Zhi 2022 | Determining the factors associated with oral health-related quality of life in Chinese elders: Findings from the fourth national survey | Community dentistry and oral epidemiology | Epidemiological study |
| Zuluaga 2012 | Oral health in institutionalised elderly people in Oslo, Norway and its relationship with dependence and cognitive impairment | Gerodontology | Epidemiological study |
